# Structural Basis for TatA Oligomerization: An NMR Study of *Escherichia coli* TatA Dimeric Structure

**DOI:** 10.1371/journal.pone.0103157

**Published:** 2014-08-04

**Authors:** Yi Zhang, Yunfei Hu, Hongwei Li, Changwen Jin

**Affiliations:** 1 Beijing Nuclear Magnetic Resonance Center, Peking University, Beijing, China; 2 College of Life Sciences, Peking University, Beijing, China; 3 College of Chemistry and Molecular Engineering, Peking University, Beijing, China; 4 Beijing National Laboratory for Molecular Sciences, Peking University, Beijing, China; Centre National de la Recherche Scientifique, Aix-Marseille Université, France

## Abstract

Many proteins are transported across lipid membranes by protein translocation systems in living cells. The twin-arginine transport (Tat) system identified in bacteria and plant chloroplasts is a unique system that transports proteins across membranes in their fully-folded states. Up to date, the detailed molecular mechanism of this process remains largely unclear. The *Escherichia coli* Tat system consists of three essential transmembrane proteins: TatA, TatB and TatC. Among them, TatB and TatC form a tight complex and function in substrate recognition. The major component TatA contains a single transmembrane helix followed by an amphipathic helix, and is suggested to form the translocation pore via self-oligomerization. Since the TatA oligomer has to accommodate substrate proteins of various sizes and shapes, the process of its assembly stands essential for understanding the translocation mechanism. A structure model of TatA oligomer was recently proposed based on NMR and EPR observations, revealing contacts between the transmembrane helices from adjacent subunits. Herein we report the construction and stabilization of a dimeric TatA, as well as the structure determination by solution NMR spectroscopy. In addition to more extensive inter-subunit contacts between the transmembrane helices, we were also able to observe interactions between neighbouring amphipathic helices. The side-by-side packing of the amphipathic helices extends the solvent-exposed hydrophilic surface of the protein, which might be favourable for interactions with substrate proteins. The dimeric TatA structure offers more detailed information of TatA oligomeric interface and provides new insights on Tat translocation mechanism.

## Introduction

Distinct to the ubiquitous Sec-pathway that translocates proteins across lipid membranes in unfolded states via a threading mechanism, the twin-arginine transport (Tat) system translocates proteins across membranes in fully folded states, some in homo- or hetero-oligomeric forms, or harboring cofactors or metal centers [1], [2]. In consequence, transportation of these folded proteins across membranes would require a highly dynamic and versatile translocation pore. Thus far, the molecular mechanism for Tat system to carry out the challenging task of translocating proteins with various shapes and sizes remains unclear.

The *Escherichia coli* Tat system is a model system that has been extensively investigated. It contains three essential components: two single-pass transmembrane proteins TatA and TatB, and a six-pass transmembrane protein TatC. Among these, TatA is the major component and is suggested to form the protein translocation pore by self-oligomerization [3]–[5]. Experimental evidences suggest that the TatB and TatC components function as a complex in substrate recognition and binding [6]–[8]. In the presence of proton motive force (PMF), the substrate-binding event triggers the recruitment of TatA subunits, which subsequently assemble into a high-oligomeric state for substrate translocation [9]. The high-oligomeric TatA has been suggested to function as the protein translocation pore, and the mechanism for TatA oligomer assembly proves essential for understanding Tat-mediated protein translocation.

A number of studies have been carried out to investigate the structure and oligomerization mechanism of the TatA subunit [10]–[13]. *E. coli* TatA is an 89 amino acid protein containing one transmembrane helix (TMH) and one amphipathic helix (APH). Both *in vitro* electron microscopy (EM) [3] and *in vivo* single-molecule imaging [5] studies showed that TatA self-oligomerizes into complexes of variable sizes and form a channel-like structure. Electron paramagnetic resonance (EPR) data suggested that residues Ile12 and Val14 in the TMH are the main contributors to TatA assembly [14]. Recently, the NMR structure of a TatA T22P mutant in dodecylphosphocholine (DPC) micelles was solved [12]. This mutant exists in the monomeric form but displays a spectrum similar to the oligomeric form. Based on this structure, a 9-mer TatA oligomer was further modelled using distance restraints from both EPR and NMR results [12]. However, inter-subunit NOE restraints are sparse and limited only in the TMH segment. On the other hand, previous biochemical studies demonstrated that the TatA function is more sensitive to mutations in the APH segment than in the TMH [15]. This raises the question of how APH is involved in the oligomerization.

Herein we report the construction and structure determination of TatA dimer by solution NMR spectroscopy. The dimeric TatA is stabilized by the introduction of a disulphide cross-linkage in the N-terminus without disrupting the overall structure. By using this dimeric sample in combination with high-resolution NMR spectroscopy, we were able to observe more extensive inter-subunit contacts in not only the TMH region, but also the APH region. The dimeric TatA structure offers a more detailed atomic picture of TatA oligomerization interface and provides valuable clues for further investigations.

## Materials and Methods

### NMR Samples Preparations

Two TatA constructs MCG-TatA and MCG-TatA_1–55_ were prepared, both containing three additional amino acids Met-Cys-Gly at the N-terminus. In the MCG-TatA_1–55_ construct, the C-terminal region was truncated and only residues Met1-Gln55 were kept. Expression and purification procedures of TatA proteins were similar to previously reported [10]. TatA protein was purified in DPC (Anatrace) and exchanged to a buffer containing 50 mM sodium phosphate (pH 7.0). For N-terminal linked dimeric TatA samples, purified MCG-TatA or MCG-TatA_1–55_ was first incubated with 10 mM H_2_O_2_ for 10 minutes at room temperature, followed by ultrafiltration to remove the oxidant. For ^13^C/^15^N-filtered-NOESY experiments, the sample was prepared by mixing unlabeled and ^13^C/^15^N-labeled MCG-TatA at 2∶1 molar ratio, then treated with 20 mM DTT in ∼300 mM deuterated DPC (dDPC, Anatrace). Subsequently, DTT and dDPC concentrations were gradually reduced by ultrafiltration. The mixed protein sample was then treated with H_2_O_2_ as described above. In the final sample, the detergent-to-protein molar ratio (DPR) was about 80 (Table S1 in File S1).

For paramagnetic relaxation enhancement (PRE) experiments [16], we constructed two TatA mutants TatA_1–55_-I12C and TatA_1–55_-I11C. Unlabeled TatA_1–55_-I12C or TatA_1–55_-I11C protein (0.5 mM) was dissolved in 50 mM sodium phosphate buffer (pH 7.0) with 150 mM DPC. 1-oxy-2,2,5,5-tetramethyl-D-pyrroline-3-methyl methanethiosulfonate (MTSL, Toronto Research Chemicals, Inc.) was added to a final concentration of 5 mM. The sample was mixed with 0.5 mM ^15^N-labeled TatA_1–55_ protein and incubated overnight at room temperature. Excess MTSL and DPC were removed by buffer exchange. The final DPR was 40. To obtain the diamagnetic reference spectra, MTSL was reduced by the addition of 4 µL of a 500 mM ascorbic acid solution.

### NMR Spectroscopy

The NMR experiments were performed at 35°C on Bruker Avance 600, 700 and 800 MHz spectrometers equipped with four RF channels and triple-resonance cryo-probes with pulsed-field gradients. The chemical shift assignments were obtained by conventional triple resonance experiments [17] using ^13^C/^15^N-labeled TatA sample in dDPC micelles. 3D ^15^N- and ^13^C-edited NOESY-HSQC spectra (mixing times 100 ms and 200 ms) for TatA in dDPC micelles were collected to confirm the assignments and obtain distance restraints. For N-terminal linked dimeric TatA comprising mixed unlabeled and ^13^C/^15^N-labeled subunits, 3D ^13^C/^15^N-filtered ^13^C-edited NOESY-HSQC experiments (mixing time of 200 ms, 300 ms and 400 ms) and ^13^C-edited NOESY-HSQC experiment (mixing time of 150 ms) were recorded to obtain intermolecular NOEs.

### RDC Measurements

The backbone N-H RDC measurements of *E. coli* TatA were performed using the liquid crystalline phase of G-tetrad DNA [18]. ^1^H-^15^N IPAP-HSQC spectra were collected for the weakly aligned and the isotropic samples at 35°C, and the RDCs were extracted from the differences in ^1^H-^15^N splitting [19]. The data were analyzed using software packages PALES [20] and MODULE [21].

### Structure Calculation of TatA

The structure calculation of *E. coli* TatA was carried out using inter-proton NOE-derived distance restraints and dihedral angle restraints. The C-terminal tail (K49-V89) is largely unstructured based on NOESY spectra analysis and was excluded during the calculation. The program TALOS was used to predict dihedral angles ψ and φ restraints [22].

Calculations of monomeric and dimeric TatA structures were carried out using the program Xplor-NIH [23] with or without intermolecular NOEs following a procedure similar to previously described [10]. For TatA monomer, 100 structures were calculated in each cycle, and 50 conformers with the lowest total energy were selected for fitting with backbone N-H RDCs using the single value decomposition method by the program PALES [20]. In the final cycle, 29 conformers showed correlation coefficient values higher than 0.90. Among these, 20 conformers with the lowest energy were selected as the representative structures. The calculation for TatA dimer was similar as above. In the final cycle, 10 lowest energy conformers with correlation coefficient values higher than 0.80 were selected to represent the dimeric structure. The atomic coordinates of monomeric and dimeric TatA have been deposited in the Protein Data Bank with accession code PDB 2MN7 and 2MN6 respectively.

### Backbone Dynamics

The backbone ^15^N relaxation parameters including the longitudinal and transverse relaxation rates *R_1_* and *R_2_* were determined for both the wild-type TatA sample at high DPC concentration (DPR of 200) and TatA_1–55_ sample at low DPC concentration (DPR of 40). The experiments were performed on a Bruker Avance 800-MHz NMR spectrometer at 35°C. For the wild-type TatA sample at high DPC concentration, the delays used for the *R_1_* experiments were 10 (×3), 100, 200, 300, 400, 600, 800, 1,000, 1,600, 2,400, 3,200 and 4,000 ms, and those used for the *R_2_* experiments were 7.4 (×2), 14.8, 22.3, 29.7, 37.1, 52.0, 66.8, 89.0, 111.4 and 133.6 ms. For TatA_1–55_ sample at low DPC concentration, the delays used for the *R_1_* experiments were 10 (×2), 100, 300, 600, 1,000, 1,600, 2,400 and 3,200 ms, and those used for the *R_2_* experiments were 7.4 (×2), 14.8, 22.3, 29.7, 52.0 and 66.8 ms. The relaxation rate constants were obtained by fitting the peak intensities to a single exponential function using the nonlinear least-squares method as described [24].

### Blue-Native PAGE Analysis

Blue-native PAGE of wild-type TatA and MCG-TatA was performed as described [25]. For this experiment, the TatA proteins were purified by Ni(II)-affinity chromatography similarly as the NMR sample, except that the detergent C12E9 (Anatrace) was used instead of AZ314 (Anatrace) and DPC. Subsequently, the purified protein was subjected to gel filtration on a Superdex 200 column using an ÄKTA FPLC system (Amersham Biosciences) in 20 mM MOPS, 200 mM NaCl, 0.1% C12E9, pH 7.2. Purified proteins were separated on a 5%–14% acrylamide gradient gel and stained by Coomassie brilliant blue.

### Functional Complementation Assay

*In vivo* functional assay was carried out using the *E. coli tatA* gene knockout strain (CGSC: JW3813-1). The genes of wild type TatA (wt-TatA), MCG-TatA and a truncated version TatA_1–55_, were constructed into pET-21a plasmids (Novagen) and transformed into the *ΔtatA* strain. The sodium dodecyl sulfate (SDS) resistance phenotype of TatA variants was tested on LB agar plates containing 2% SDS [26]. All *ΔtatA* strains carrying plasmids with *tatA* variant genes were first grown in LB media with ampicillin and kanamycin at 35°C until OD_600_ reached 0.6 and induced with 20 mg/L isopropyl-β-D-thiogalactoside (IPTG) for 2 h. The culture was diluted to OD_600_ of 0.2 and tenfold dilution series were spotted on agar plates. The plates were anaerobically incubated at 35°C using an AnaeroPack system (Mitsubishi Gas Chemical, Tokyo, Japan) for 72 hours.

## Results

### Characterization of the Oligomeric State of TatA

The *E. coli* TatA protein was first characterized under a relatively high DPR of 300. Under this experimental condition, the sample shows a unique set of cross peaks in the ^1^H-^15^N heteronuclear single quantum coherence (HSQC) spectrum. By using ^13^C/^15^N-uniformly labeled TatA protein solubilized in dDPC micelles, we were able to obtain near complete backbone and side chain assignments for the full-length protein (Figure S1 in File S1). Secondary structural analysis by consensus chemical shifts index (CSI) demonstrates that the protein comprises two helices in the N-terminal region and an unstructured C-terminus (Figure S2 in File S1) [27], [28], which is in accordance with previously reported TatA structures [10], [12].

During sample preparation and optimization, we observed that changes in DPR would affect the oligomerization state of TatA. At a high DPR (>200), the HSQC spectrum shows one set of cross-peaks. When the DPR decreases, new sets of cross peaks appeared, while intensities of the original set gradually decreased (see Supporting Results and Discussion and Figure S3 in File S1 for more details). The appearance of new peaks was observed for residues in the TMH, the hinge region, as well as the N-terminal part of APH (residues Leu25-Gly33, designated as ‘proximal APH’ hereafter following a previous publication by Rodriguez *et al* [12]), suggesting these regions exist in two oligomeric states that are in slow exchange with each other. The observation that TatA oligomerization affects not only the TMH but also the APH region is consistent with the previous observation of a truncated version of TatA (residue 1–49) [12]. On the other hand, residues in the C-terminal part of APH (residues Ala34-Ser44, designated as ‘distal APH’ hereafter following a previous publication [12]) only show chemical shift perturbations but not appearance of new peaks, whereas the unstructured C-terminus is unaffected by reducing the DPR. For clarity, we designated the original set of cross peaks as the 1^st^ set, and the new set of peaks as the 2^nd^ set.

**Figure 1:**
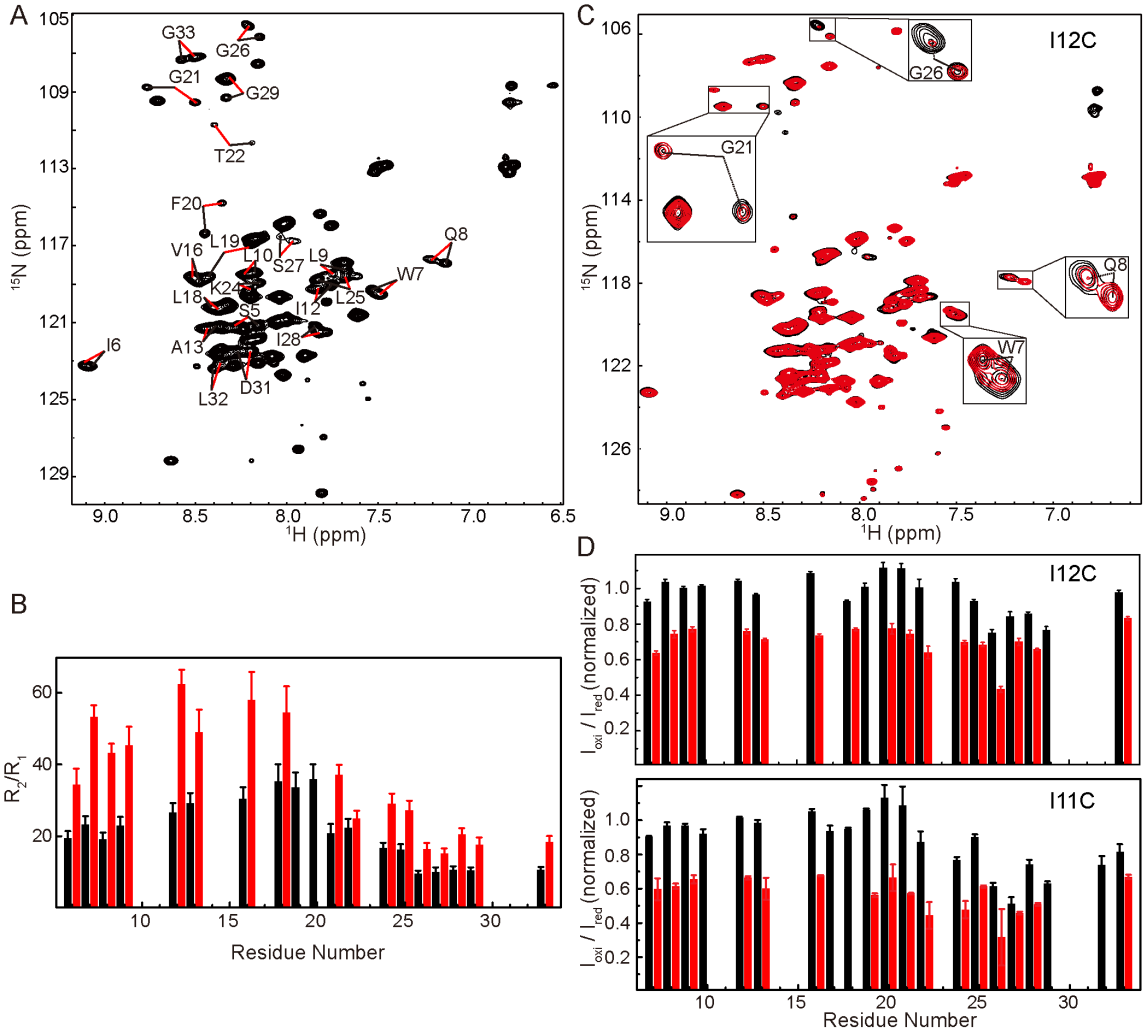
Characterization of the 2^nd^ set of peaks. A) ^1^H-^15^N HSQC spectrum of TatA_1–55_ containing two sets of peaks at DPR of 70. Representative residues are labeled with black and red lines pointing to the 1^st^ and 2^nd^ sets of peaks, respectively. B) The backbone ^15^N relaxation parameter *R_2_*/*R_1_* of TatA_1–55_ as a function of residue numbers. The data were collected at DPR of 70. Black and red bars represent the 1^st^ and 2^nd^ sets of peaks, respectively. Only residues showing two sets of separable peaks were analyzed. C) ^1^H-^15^N HSQC spectrum of ^15^N-labeled TatA_1–55_ mixed with equal molar of unlabeled TatA_1–55_-I12C-MTSL. The reference spectrum and the spectrum with PRE effect are shown in black and red, respectively. The data were collected at DPR of 40. D) Intermolecular PRE effect on the two sets of peaks using TatA_1–55_-I12C-MTSL or TatA_1–55_-I11C-MTSL. Black and red bars represent the 1^st^ and 2^nd^ sets of peaks, respectively. The data were collected at DPR of 45. The peak intensity ratios were normalized using the well-resolved and generally unaffected peaks from the 1^st^ set for both (C) and (D).

To overcome the problem of spectral overlap, we generated a truncated TatA mutant to remove the unstructured C-terminal region, which has been shown to be unessential for TatA function [29]. This mutant contains only residues Met1-Gln55, and is designated as TatA_1–55_ hereafter. To better characterize the oligomeric property of TatA, a TatA_1–55_ sample was prepared in which the DPC concentration adjusted to an appropriate level (DPR ∼70) so that the ^1^H-^15^N HSQC spectrum contains two sets of peaks (Figure 1A). Backbone ^15^N longitudinal and transverse relaxation rates were measured, and the *R_2_/R_1_* ratios were calculated to analyze the oligomerization states of the two sets of peaks. As shown in Figure 1B, the average *R_2_/R_1_* ratio of residues on the TMH region is 26 for the 1^st^ set of signals, and 52 for the 2^nd^ set. The data of the 1^st^ set correlates well with a protein-micelle complex showing an average diameter of ∼51 Å, which can be fitted to a monomeric transmembrane helix surrounded by ∼54 DPC molecules. The *R_2_/R_1_* ratio of the 2^nd^ set corresponds to a diameter of 57 Å, which fits well to a dimeric model showing a ∼3 Å increase in radius (see Supporting Results and Discussion and Figure S6 in File S1 for more details).

### Construction of a Stable TatA Dimer

To verify that the 2^nd^ set of peaks corresponds to a dimeric form of TatA, we mutated residue Ile12 to cysteine in the TatA_1–55_ construct, and tagged the cysteine residue with MTSL for paramagnetic relaxation enhancement (PRE) analysis. For clarity, we abbreviate this sample as TatA_1–55_-I12C-MTSL hereafter. We then mixed equal molar of unlabeled TatA_1–55_-I12C-MTSL with ^15^N-labeled TatA_1–55_, and optimized the DPC concentration so that the HSQC spectrum contains two sets of peaks. The PRE effect was obtained by comparing the peak intensities in the PRE and reference spectra (see Materials and Methods). Our results showed that the inter-molecular PRE effect is evident on the 2^nd^ set of peaks, while the 1^st^ set is scarcely perturbed (Figure 1C, 1D). An experiment using the I11C mutation gave similar results (Figure 1D). These observations suggest that the 2^nd^ set of peaks represents a condition in which two TatA subunits are closer in space, such as two TatA monomers trapped in one micelle. For the PRE experiment using the TatA_1–55_-I12C-MTSL sample, the general intensity reduction for the 2^nd^ set of peaks is about 25%, which is in good agreement with the expected result based on a dimeric model, but not a trimetric or a tetrameric model (see Supporting Results and Discussion and Figure S8 in File S1 for more details). The observation that the PRE effect induced by the I11C mutation appears more significant than the I12C mutation could be explained by the fact that Ile11 is not located on the dimerization interface and the flexibility of the attached paramagnetic tag allows more populations to be affected (Figure S8 in File S1).

**Figure 2:**
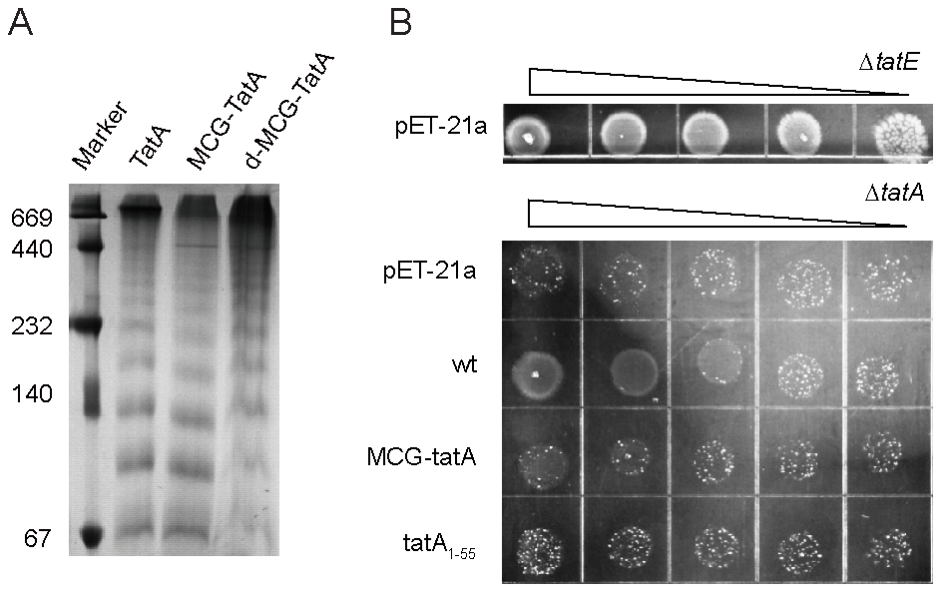
Characterization of the activity of MCG-TatA. A) Blue-native PAGE of wt-TatA, MCG-TatA and d-MCG-TatA. The mutant proteins retain the characteristic ladder pattern as the wild-type TatA. The molecular masses (kDa) of marker proteins are given on the left. B) Tenfold serial dilution of *ΔtatA* mutant strain expressing TatA variants on SDS-containing medium. The plates were anaerobically incubated at 35°C for 72 hours and then photographed (see more details in Materials and Methods). The upmost panel corresponds to a positive control experiment using the *ΔtatE* mutant strain transformed with empty pET-21a plasmids. The lower panel corresponds to the *ΔtatA* mutant strain transformed with either empty pET-21a plasmids or plasmids carrying wt-TatA gene or mutated variants.

Finally, for preparation of a stabilized TatA dimer, we constructed a mutant protein in which the N-terminus of wild-type TatA was extended by three residues Met-Cys-Gly, named as MCG-TatA. This mutant protein was cross-linked together by a disulfide bond under oxidizing condition, generating a covalently linked TatA dimer, abbreviated as d-MCG-TatA (‘d’ designates dimer). The introduction of a disulfide bond at the flexible N-terminus brings two TatA molecules close together, while at the same time avoids perturbation at structured regions. The formation of the TatA dimer was validated by SDS-PAGE as well as NMR spectroscopy (Figure S9 in File S1). In addition, the formation of a disulfide bridge is confirmed by the Cβ chemical shift at ∼41 ppm for the cysteine residue in the tripeptide extension. The backbone ^15^N dynamics data of d-MCG-TatA showed similar *R_2_/R_1_* values compared to the 2^nd^ peak set of TatA_1–55_ (Figure S7 in File S1). The oligomerization pattern of MCG-TatA and d-MCG-TatA were both verified to be similar to the wild-type protein by blue native gel analysis (Figure 2A).

Moreover, we used a modified SDS resistance assay to test MCG-TatA function *in vivo*. The assay was designed by combining two previously reported growth tests of *tat* deletion mutants, namely the growth in the presence of SDS or under anaerobic atmosphere [26], to enhance the phenotype of *ΔtatA* in the presence of the *tatA* gene homolog *tatE*. We discovered that the *ΔtatA* bacterial strain can grow in the presence of SDS under aerobic condition or under anaerobic in the absence of SDS, but cannot grow on SDS plates incubated anaerobically. We therefore used this system to perform the functional complementation assay of wild-type TatA and its variants. As shown in Figure 2B, MCG-TatA is able to partially restore SDS resistance in *E. coli ΔtatA* strain under anaerobic growth condition, whereas its activity is lower compared to wt-TatA. However, MCG-TatA supports the bacterial growth better than the TatA_1–55_, which was suggested to retain the functional activity of TatA as previously reported under a different experimental condition [29]. Our current results suggest that the N-terminal tripeptide extension has less effect on the TatA activity than the truncation of C-terminal residues under the severe growth condition, and indicate that the d-MCG-TatA sample can be used as a mimic for characterizing the dimeric TatA structure.

**Figure 3:**
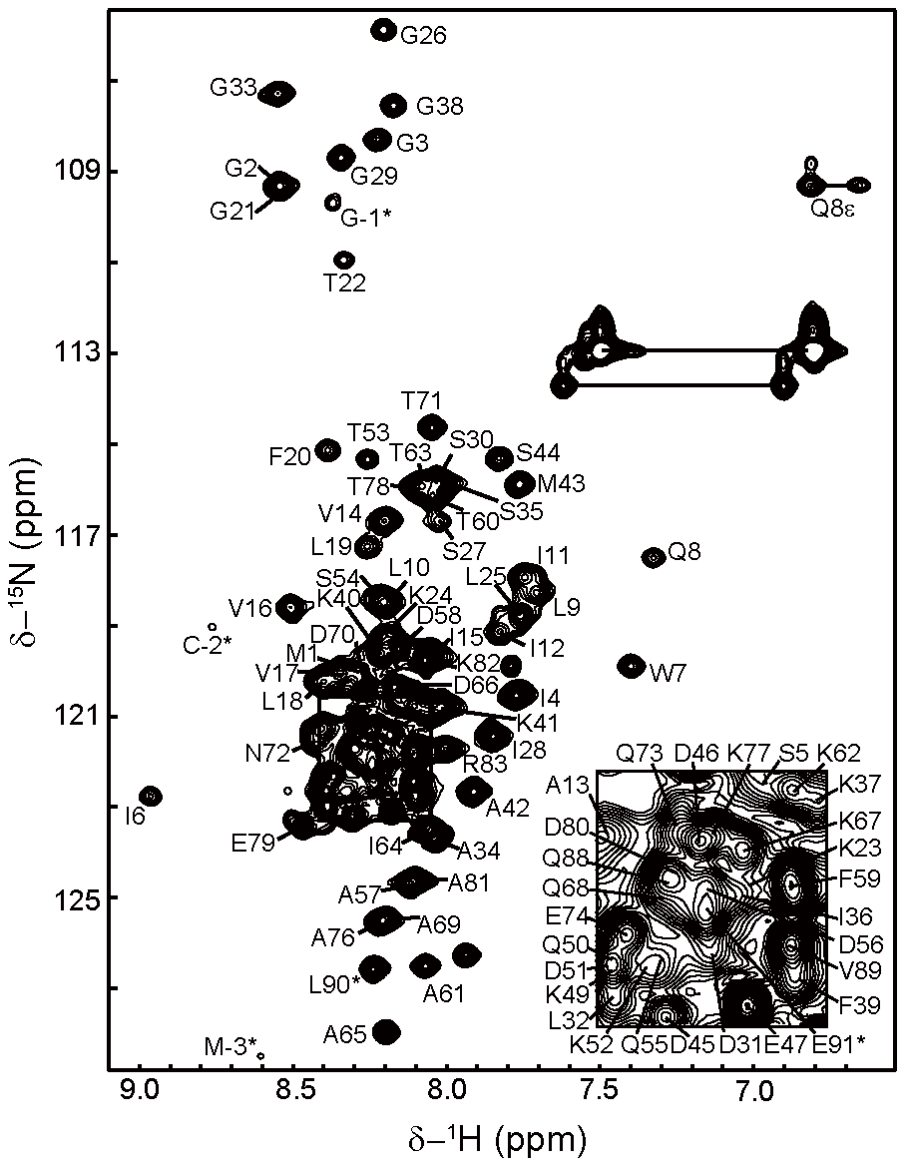
2D ^1^H-^15^N HSQC spectrum of d-MCG-TatA in DPC micelles annotated with the backbone assignments. The spectrum was collected on a Bruker Avance 800(with a cryo-probe) at 35°C and a DPR of 86. The assignments are labeled with the one-letter amino acid code and residue number. The side chain NH_2_ peaks of Asn and Gln are connected by horizontal lines. The asterisk indicates residues of the N-terminal M(-3)C(-2)G(-1) extension and the C-terminal His-tag.

Figure 3 shows the 2D ^1^H-^15^N HSQC spectrum of d-MCG-TatA in DPC micelles. Spectral comparisons between MCG-TatA, d-MCG-TatA and TatA (Figure S10 in File S1) indicate that d-MCG-TatA closely resemble the dimeric form (2^nd^ peak set) of TatA. The chemical shift assignments were obtained for 96% of backbone amides except for proline residues. In comparison with the monomeric TatA, the cross peaks corresponding to the N-terminal of TatA (residue 1–44) are broadened and show chemical shift perturbation, while those in the C-terminal unstructured region are unaffected.

### Structures of Monomeric and Dimeric TatA

Solution structures of monomeric and dimeric TatA were calculated based on NOE derived distance restraints. The atomic coordinates have been deposited in the Protein Data Bank (PDB code: 2MN7 and 2MN6). For the calculation of the dimeric structure, inter-molecular NOEs were determined by 3D ^13^C/^15^N-filtered NOESY experiments using d-MCG-TatA sample containing mixed ^13^C/^15^N-labeled and unlabeled protomers. A total of 75 unambiguous inter-subunit restraints were first identified and subsequently used to extend NOE assignments in 3D ^13^C, ^15^N-filtered-^13^C-edited NOESY and 3D ^13^C-edited NOESY spectra. Finally, 139 inter-subunit NOE restraints were identified and used in the calculation of dimeric TatA structures. Structural statistics for both monomeric and dimeric TatA are summarized in Table 1.

**Table 1:** Structural statistics for *E. coli* TatA.

Experimental restraints	monomer	dimer
Total NOE	1636	3477
Total unambiguous NOE	1067	2204
Intra-residue	451	902
Inter-residue		
Sequential (|i-j| = 1)	328	656
Medium-range (|i-j|≤4)	257	510
Long-range (|i-j|≥5)	68	136
Total Ambiguous NOE	569	1134
Intermolecular*		139
Total dihedral angle restraints (φ+ψ)	65 (32+33)	130 (64+66)
Restraint Violations		
Distance restraint violations >0.5 Å	1	4 (<0.6 Å)
Dihedral angle restraint violations >5°	0	1 (<5.5°)
RMSD** from mean structure (Å)		
All heavy atoms	1.140±0.262	1.254±0.381
All backbone atoms	0.844±0.252	1.005±0.346
Secondary structure heavy (6–20, 24–42)	1.084±0.258	1.222±0.364
Secondary structure backbone (6–20, 24–42)	0.751±0.235	0.980±0.328
Ramachandran Statistics† (%)		
Most favored regions	95.9	87.7
Additional allowed regions	3.9	12.1
Generously allowed regions	0.2	0.2
Disallowed regions	0	0

*The total intermolecular NOEs include all unique and ambiguous NOEs identified from 3D ^13^C/^15^N-filtered ^13^C-edited NOESY-HSQC experiment and ^13^C-edited NOESY-HSQC experiment.

**The RMSD values were calculated for residues 5–44.

† The values of Ramachandran statistics were calculated for residues 5–44. The C-terminal unstructured region is excluded from the analysis.

**Figure 4:**
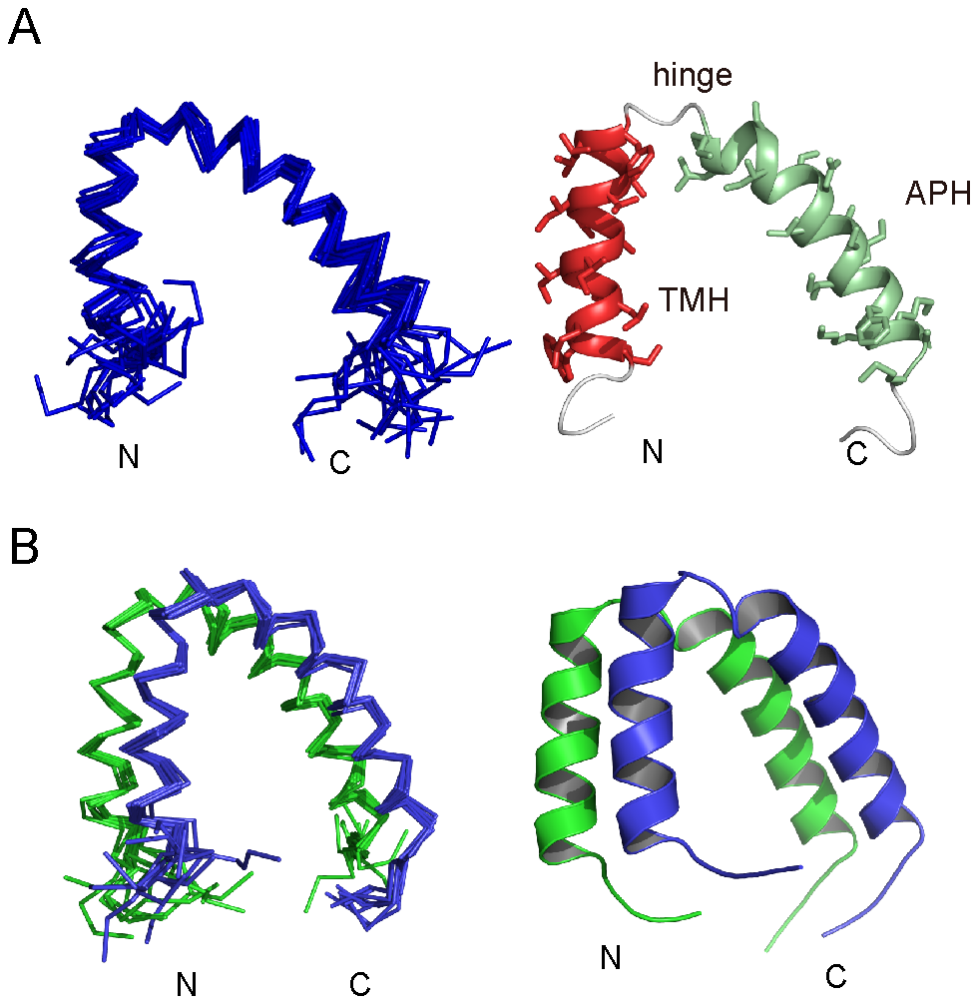
Solution structures of monomeric and dimeric TatA. A) Ensemble (left) and cartoon (right) representations of monomeric *E. coli* TatA structure. B) Ensemble (left) and cartoon (right) representations of dimeric *E. coli* TatA structure.

As shown in Figure 4A, the structure of the monomeric *E. coli* TatA consists of a 15-residue TMH (residues Ile6-Phe20), a 21-residue APH (residues Lys24-Met43) and a mostly unstructured C-terminal region. The two helices are linked by a highly conserved four-residue hinge F_20_G_21_T_22_K_23_, and pack into an L-shaped structure. The structure is generally similar to the *E. coli* TatA (T22P) structure previously reported [12]. When we align the two structures using backbone atoms from all structural regions (residues Ile6-Met43), the derived root mean square deviation (RMSD) of the Cα atoms is 2.5 Å. However, when aligning the TMH and APH separately, higher structural convergence is observed with Cα RMSD values of 0.6 Å and 1.6 Å, respectively. The subtle difference between the two structures resides in the relative orientation between the two helices and the kink at Gly33, which may be due to the one-residue mutation or differences in sample condition. Despite this variance, similar interaction network was observed among residues Val16, Val17, Phe20, Thr22/Pro22 and Leu25 at the hinge between the two helices.

As shown in Figure 4B, the TatA dimer is formed by the side-by-side packing of two TatA subunits. The structures of the two individual protomers are essentially similar to the monomeric TatA structure. The characteristic α-helical NOE patterns are preserved in both TMH and APH. The intra-molecular NOE contacts between the two helices, in particular the interaction network at the hinge region are the identical in monomeric and dimeric TatA. Furthermore, the RDC data of the d-MCG-TatA sample fit reasonably well with both the monomeric and dimeric structures, with correlation coefficient values *R* of 0.88 and 0.81 respectively, suggesting no significant changes in the TMH-APH orientation upon dimerization.

### The TatA Oligomerization Interface

In the dimeric TatA structure, inter-subunit NOEs are observed for both the TMH and APH segments. Among these, two-thirds are contributed by residues in the TMH. The number of inter-subunit NOEs observed in the filtered-NOESY spectrum for residues in the APH is about half of that for the TMH, and the intensities of the NOE peaks for the APH are generally weaker than those in the TMH (∼40%). In addition, the NOE peaks for residues in the distal APH are weaker than those in the proximal APH. Considering the fact that residues in the distal APH show only peak shifts during the DPR-dependent TatA oligomerization as presented above, a possible scenario is that the TMH interactions are stronger and play a main role in TatA dimer formation, whereas the APH interactions are more dynamic and undergo fast exchanges between ‘contact’ and ‘off-contact’ states (Figure S12 in File S1). Moreover, residues in the hinge region and residue Gly33 in the APH show relatively weak intramolecular NOE signals possibly due to conformational exchanges. We were therefore unable to detect intermolecular NOEs for these residues although they reside on the dimeric interface.

**Figure 5:**
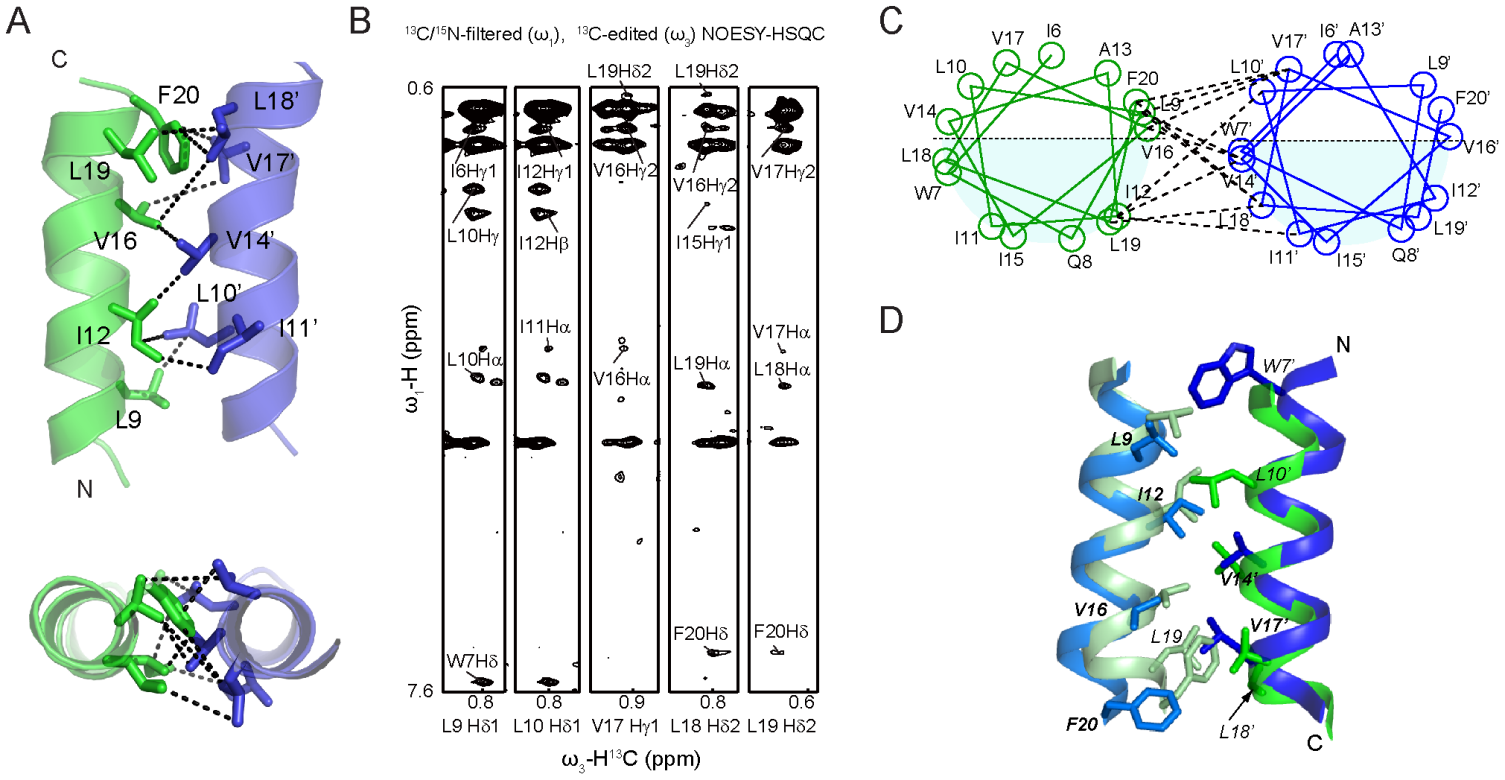
Interface of TMH regions in dimeric TatA. A) Dimerization interface between the TMH segments of two subunits. Only TMH segments are shown for clarity. The omitted APHs are positioned below the paper in the upper panel. B) Representative strips from ^13^C/^15^N-filtered, ^13^C-edited NOESY experiment using a d-MCG-TatA sample with a mixture of uniformly ^13^C/^15^N-labeled and unlabeled MCG-TatA (mixing time 300 ms). The experiment selectively detects inter-proton NOEs between a proton attached to a ^13^C-labeled carbon (the ω_3_ dimension) and protons attached to unlabeled carbon or nitrogen atoms (the ω_1_ dimension). All protons attached to ^13^C/^15^N-isotopes are filtered out in the ω_1_ dimension. Therefore only the intermolecular NOEs between a uniformly labeled subunit and an unlabeled subunit are retained, whereas intramolecular NOEs or intermolecular NOEs in homo-labeled or homo-unlabeled dimers do not show up. C) Structure-based helical wheel diagrams of the dimer interface, with representative NOE interactions shown in dashed lines. Positively and negatively charged residues are shown in blue and red, respectively. The helical wheel diagram was drawn using an in-house written script based on the atomic coordinates of the dimeric TatA structure. D) Structural comparison of the TMH dimerization interface. The dimeric TatA structures determined in the present study are shown in green and pale green. The atomic coordinates of two adjacent subunits are subtracted from 9-mer TatA structure (PDB 2LZS) and shown in blue and sky blue. Side chains observed to show inter-molecular NOEs are shown in sticks and labeled.

The interface between two TMHs is formed by residues Leu9, Ile12, Val16 and Leu19 from one subunit, and residues Leu10, Val14, Val17 and Leu18 from the other (Figure 5A, 5C). Residues Ile12 and Val14 are located in the center of the TMH contacting surface and are close to each other, which is consistent with previous EPR observations [14]. In a recent NMR study of the *E. coli* TatA structure, a 9-mer TatA complex was modeled based on the monomeric TatA-T22P mutant structure [12]. To compare the dimerization pattern, we extracted two adjacent protomers from the 9-mer TatA complex (PDB: 2LZS) and aligned it with our dimeric structure. The interface between two TMHs is generally identical between the two structures, which is comprised mainly of hydrophobic residues Leu9, Ile12, Val14, Val16, Leu18 and Phe20 (Figure 5D). Most residues identified to display inter-subunit NOE signals, including Leu9, Ile12, Val14, Val16, Leu18 and Phe20, are the same in both structures. However, the two structures display subtle difference in the relative orientations between neighboring TMHs. This could probably be attributed to the different procedures used for building the models. It is also possible that the TMH orientations could be fine-tuned in correspondence to TatA oligomers of various sizes.

**Figure 6:**
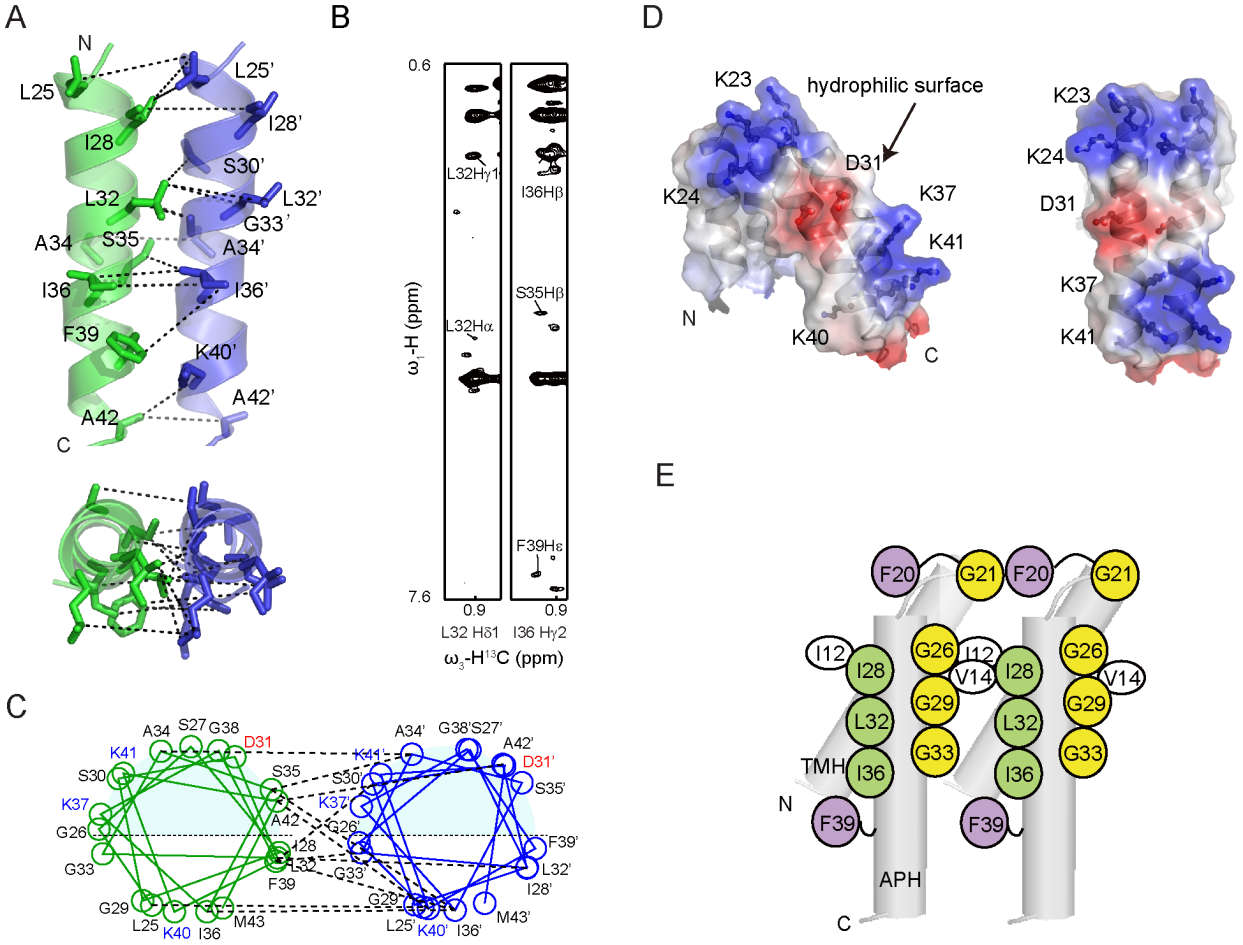
Interface of APH regions in dimeric TatA. A) Dimerization interface between the APH segments of two subunits. Only APH segments are shown for clarity. The omitted TMHs are positioned below the paper. B) Representative strips from ^13^C/^15^N-filtered, ^13^C-edited NOESY experiment using a d-MCG-TatA sample with a mixture of uniformly ^13^C/^15^N-labeled and unlabeled MCG-TatA (mixing time 300 ms). The experiment selectively observes intermolecular NOEs as described in Figure 5. C) Structure-based helical wheel diagrams of the dimer interface, with representative NOE interactions shown in dashed lines. Positively and negatively charged residues are shown in blue and red, respectively. The helical wheel diagram was drawn using an in-house written script based on the atomic coordinates of the dimeric TatA structure. D) Surface representation of the TatA dimer with electrostatic potential. Side chains of charged residues are shown in sticks and labeled. E) A schematic summary of important residues at the TatA oligomerization surface.

On the other hand, we also identified inter-subunit NOEs in the APH segment by using ^13^C/^15^N-filtered NOESY experiments. In contrast to the TMH, inter-APH NOE cross-peaks are generally weaker, and have not been observed in the previous studies [12]. The inter-APH NOE contacts are observed between residues Ile28, Leu32, Ser35, Ile36 and Phe39 from one subunit, and residues Leu25, Ser30, Gly33, Ala34, Lys37 and Lys40 from the other (Figure 6A-C). Among these, the long-chain hydrophobic residues Ile28, Leu32 and Ile36 are aligned on one side of the APH, forming a highly hydrophobic strip. On the opposite side of the helix, three glycine residues (Gly26, Gly29 and Gly33) align into another strip. These two strips of residues pack side by side at the dimeric interface (Figure 6E).

Functionality of TatA has been demonstrated to crucially depend on the APH segment, since many cysteine substitutions abolished Tat activity [15], [30], [31]. These mutations may affect either TatA self-assembly or TatA-substrate interactions. In particular, single point mutations at the dimeric interface including G29C, G33S, G33A, G33D, I28T, L32C, I36T and I36C have been reported to abolish the Tat function [15], [31], [32], whereas non-charged residues such as Ser27, Ala34 and Ser35 that do not contribute to the APH assembly appear non-essential. Moreover, cross-linking experiments have shown that G33S and G33D mutations significantly affect TatA self-assembly. The G33D mutation showed no cross-linking activity at all and an electrostatic repulsion by this mutation was suggested [32].

### The Hydrophilic Surface is Extended upon Dimerization

The structure of TatA shows a clear segregation of hydrophobic and hydrophilic residues. The TMH segment is highly hydrophobic. The APH segment contains a relatively hydrophobic side that faces the TMH and a hydrophilic side that is exposed to the aqueous solution. The exposed hydrophilic surface comprises most of the charged residues including Ser27, Ser30, Asp31, Lys37 and Lys41 from APH, as well as residues Lys23-Lys24 from the hinge. In the dimeric form of TatA, the hydrophilic surfaces of two protomers are positioned side-by-side, and form a larger highly charged area as shown in Figure 6D. This area displays an electrostatic potential pattern with negative charges in the center, sandwiched by two positively charged zones.

Previous functional studies suggested that a charged surface may be essential for translocation of well-folded proteins, since cysteine mutations of Lys23, Lys24, Asp31, Lys37 and Lys40 or glutamine substitutions of three lysine residues (Lys37, Lys40 and Lys41) all abolished protein transport by the Tat system [15]. A possible scenario is that upon formation of higher oligomeric states, this charged surface is more significantly extended, and better facilitate interactions with substrate proteins that are fully folded and contain generally hydrophilic surfaces.

## Discussion

The mechanism of TatA self-assembly and its role in protein transport has long been the center of research interest. Two major models for TatA mechanism have been proposed based on a variety of biochemical, structural and theoretical data emerged in recent years, the observations and interpretations of which are sometimes controversial [33]. The ‘trap-door’ or ‘pore-forming’ model that gained earlier popularity is based on the observed pore structures by cryo-EM and the possibility of APH to flip through the lipid bilayer, suggesting a protein translocation pore with hydrophilic interior formed by the APH [3], [34]. The ‘membrane-weakening’ model suggests TatA does not form pores of different sizes but aggregate to disrupt local lipid-bilayer structures, resulting in the thinning of the lipid and facilitating protein translocation [35]. This model become more favorable recently, and was supported by a number of experimental evidences [11], [36]. In both models, although the role of TMH in mediating TatA oligomerization is better characterized by multiple experimental techniques [12], [14], the role of the APH region in TatA assembly remains less well understood.

The NMR and molecular dynamics studies by Rodriguez *et al* support the ‘membrane-weakening’ model [12]. In their 9-mer TatA structure, the APH was placed on the outer side of the TMH ring. A model was postulated in which the APH contacts the substrate protein and is pulled into the lipid bilayer during translocation so that the angle between TMH and APH increases. Although no APH interactions were observed in the 9-mer TatA structure, as the number of TatA subunits increases and as the APH is pulled into the bilayer, the distance between neighboring APH becomes closer. At this stage, the weak and dynamic interaction between adjacent APHs may come to play an important role in holding multiple APHs together to form a continuous and charged surface. This could increase the interaction surface between TatA complex and fully-folded substrate proteins, and may help in substrate protein quality control or sealing of the pore to prevent leakage of small molecule, but remains to be further investigated.

Recently, Walther *et al* introduced a ‘charge zipper mechanism’ by a simulated model in which the APH and the unstructured charged C-terminus form complementary charge interactions to allow TatA assembly [37]. This model was primarily based on *Bacillus subtilis* TatA_d_ structure, and is more in line with the ‘pore-forming’ model. The APH segments, however, are not in close contact with each other based on this model. Moreover, the primary sequences of TatA from *E. coli* and other Gram-negative bacteria are somewhat different from the Gram-positive *B. subtilis* TatA sequences. In particular, the highly conserved negative charges that were suggested to be important for this ‘charge zipper’ is located immediately after the last turn of the APH in *E. coli* TatA sequence (residues D_45_D_46_E_47_), whereas in *B. subtilis* TatA_d_ and TatA_y_ sequences the negatively charged motifs are separated from the APH by a 4 or 6-residue loop. In the absence this connecting loop, it would be difficult for the negatively charged sequence of *E. coli* TatA to fold back and form the zipper conformation without unwinding the last turn of the APH. On the other hand, mutagenesis studies demonstrated this charged D_45_D_46_E_47_ sequence is essential for TatA function, whereas its exact role remains unclear [38]. In our current study, we observed intermolecular contact between two APHs, but not between APH and the charged C-terminus. However, one intriguing observation is that the truncation of the C-terminal region appears to promote DPC-dependent aggregation of TatA, suggesting a negative regulation on TatA oligomerization by the C-terminus, which remains to be further investigated.

## Conclusion

In summary, by using a combination of state of the art solution NMR and protein engineering techniques, we constructed a stable TatA dimer and observed more extensive inter-subunit contacts in not only the TMH region, but also the APH region. The dimeric TatA structure offers a more detailed atomic picture of TatA oligomerization interface and provides clues for further investigation.

## Supporting Information

File S1
**Supporting results and discussion, Supporting Table S1, and Supporting Figures S1-S12.** **Figure S1, 2D 1H-15N HSQC spectrum of TatA.** 2D 1H-15N HSQC spectrum of TatA in DPC micelles annotated with the backbone assignments. The spectrum was collected on a Bruker Avance 800 MHz spectrometer (with a cryo-probe) at 35°C. The assignments are labeled with the one-letter amino acid code and residue number. The side chain NH2 peaks of Asn and Gln are connected by horizontal lines. The asterisk indicates residues from the His-tag. **Figure S2, Secondary structures of** ***E. coli*** **TatA.** Secondary chemical shifts for 13Cα, 13Cβ, 13C′ and 1Hα resonances versus residue numbers and the consensus chemical shift index (CSI) plot of TatA in DPC micelles. The values of CSI for β-strand, α-helix and random coil are 1, -1 and 0, respectively. **Figure S3, DPR-dependent spectral changes of full-length TatA.** (A) The protein sequence of TatA is shown at the top to show the region displaying DPR-dependent chemical shift perturbations (underlined) and residues that have multiple sets of peaks (colored in red). (B) 1H-15N HSQC spectrum of full-length TatA at DPR 300 (black) and 20 (red) with dashed lines indicating the signals from the same residues. The population ratios of different conformers were calculated based on peak volumes (V1∶V2∶V3). (C) The composite chemical shift differences between the two peak sets calculated using the formula 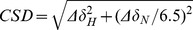. The red bars represent the residues with multiple sets of signals and the CSD was calculated between the 1st and 2nd peak sets. The black bars represent residues that show only chemical shift perturbations. Grey background represents residues that could not be analyzed. **Figure S4, Characterization of TatA at very low DPR.** 1H-15N HSQC spectrum of full-length TatA at DPR of 300 (black) and an extremely low DPR (<20, red). Representative residues are labeled with black and red lines. **Figure S5, Characterization of TatA1-55 at DPR of 30.** (A) 1H-15N HSQC spectrum of TatA1-55 at DPR of 30 with dashed lines indicating the signals from the same labeled residues. The population ratios of different conformers were calculated based on peak volumes. (B) Chemical shift perturbations between the 1st and 3rd peak sets of residues in the hinge region. Open and filled bars correspond to the chemical shift changes of backbone 15N and 1HN atoms, respectively. **Figure S6, Estimation of the TatA-DPC complexes sizes based on backbone dynamics.** The upper panel shows the 2D illustrations of back-calculated sizes of TatA-DPC complexes based on the 1st (upper left) and 2nd sets of peaks (upper right). The data of the 1st set corresponds well to a monomeric model (upper left), whereas the 2nd set exhibits a 3 Å increase in radius compared to the 1st set. The lower panel shows schematic models of a dimeric TatA-DPC complex and a tetrameric TatA-DPC complex. The averaged radius contributed by THM is estimated to be 8.3 Å or 12 Å based on the dimeric or tetrameric models, and would result in an apparent radius increase of 2.8 Å and 6.6 Å respectively. The diameter of a single helix is assumed to be 11 Å in all calculations. The experimental data of the 2nd set fits better to the dimeric model. **Figure S7, Backbone dynamics of d-MCG-TatA1-55.** The backbone ^15^N relaxation parameter *R_2_*/*R_1_* values of d-MCG-TatA_1–55_ at DPR of 100 (filled circle) in comparison with full-length TatA (open circle). The N-terminal MCG-tripeptide extension is numbered as residue -3, -2 and -1 respectively. Residues in the C-terminus of full-length TatA are not shown. **Figure S8, Illustration of inter-subunit PRE effects of different TatA oligomerization models.** Calculation of the population distribution of different oligomeric species and inter-subunit PRE effects using dimeric, trimeric and tetrameric models for paramagnetic labeling on the inter-subunit interface (panel A, TatA1-55-I12C-MTSL) or the opposite side of APH (B, TatA1-55-I11C-MTSL). The 15N-labeled and spin-labeled samples are mixed at 1∶1 molar ratio, and the 15N-labeled sample is the only source generating observable NMR signals. Small yellow star designates the position of the MTSL label, and red and blue crosses indicate expected complete or partial signal broadening by the PRE effect. The expected signal reduction ratio is calculated for each model. The experimental results obtained at DPR ∼40 for MTSL labeling at either position 12 or 11 best correlate with the dimeric model. **Figure S9, Formation of disulfide-linked d-MCG-TatA dimer.** (A) SDS-PAGE spectra of freshly eluted d-MCG-TatA from Ni-NTA and DTT reduced MCG-TatA. Positions of dimer and monomer are labeled. (B) HSQC spectra of d-MCG-TatA and MCG-TatA showing the different positions of the residue cysteine (C-2) in the oxidized and reduced states. **Figure S10, Spectral comparisons of MCG-TatA, d-MCG-TatA and wt-TatA.** 1H-15N HSQC spectra of full-length TatA at DPR of 300 (black) and 20 (red) in comparison with MCG-TatA (DPR 110, reduced state) and d-MCG-TatA (DPR 85, oxidized state). The red and blue lines indicate peaks of d-MCG-TatA and the 2nd peak set in TatA (DPR 20), respectively. Representative peaks from the same residue are grouped together and labeled. Apart from residues 1–8 that are most significantly affected by the N-terminal extension, the other residues show chemical shift differences less than 0.04 ppm for protons and 0.5 ppm for nitrogens between d-MCG-TatA and the 2nd peak set in TatA. The primary sequences of wt-TatA and MCG-TatA are shown at the bottom. **Figure S11, Verification of inter-subunit NOEs.** Representative strips from the 3D 13C/15N-filtered (ω1), 13C-edited (ω3) NOESY-HSQC spectra (mixing time 300 ms) of uniformly labeled dimer (left panels) and mixed dimer (right panels). Assignments for inter-subunit NOE cross peaks are labeled in the right panels, whereas no signals are observable in the corresponding positions in the control sample (left panels). The strong peaks present in the control experiment (left panels) originate from detergent and water signals. **Figure S12, A schematic model of the TatA dimerization.** **Table S1, Protein samples and conditions used in this study.**
(PDF)
