# Supplementary material for: Structural Basis for TatA Oligomerization: An NMR Study of Escherichia coli TatA Dimeric Structure
Source: PLoS One. 2014 Aug 4;9(8):e103157. doi: 10.1371/journal.pone.0103157 (PMC4121141; doi:10.1371/journal.pone.0103157)
Supplement: File S1 — Supporting results and discussion, Supporting Table S1, and Supporting Figures S1-S12. Figure S1, 2D 1H-15N HSQC spectrum of TatA. 2D 1H-15N HSQC spectrum of TatA in DPC micelles annotated with the backbone assignments. The spectrum was collected on a Bruker Avance 800 MHz spectrometer (with a cryo-probe) at 35°C. The assignments are labeled with the one-letter amino acid code and residue number. The side chain NH2 peaks of Asn and Gln are connected by horizontal lines. The asterisk indicates residues from the His-tag. Figure S2, Secondary structures of E. coli TatA. Secondary chemical shifts for 13Cα, 13Cβ, 13C′ and 1Hα resonances versus residue numbers and the consensus chemical shift index (CSI) plot of TatA in DPC micelles. The values of CSI for β-strand, α-helix and random coil are 1, -1 and 0, respectively. Figure S3, DPR-dependent spectral changes of full-length TatA. (A) The protein sequence of TatA is shown at the top to show the region displaying DPR-dependent chemical shift perturbations (underlined) and residues that have multiple sets of peaks (colored in red). (B) 1H-15N HSQC spectrum of full-length TatA at DPR 300 (black) and 20 (red) with dashed lines indicating the signals from the same residues. The population ratios of different conformers were calculated based on peak volumes (V1∶V2∶V3). (C) The composite chemical shift differences between the two peak sets calculated using the formula . The red bars represent the residues with multiple sets of signals and the CSD was calculated between the 1st and 2nd peak sets. The black bars represent residues that show only chemical shift perturbations. Grey background represents residues that could not be analyzed. Figure S4, Characterization of TatA at very low DPR. 1H-15N HSQC spectrum of full-length TatA at DPR of 300 (black) and an extremely low DPR (<20, red). Representative residues are labeled with black and red lines. Figure S5, Characterization of TatA1-55 at DPR of 30. (A) 1H-15N HSQC spectrum of T [file pone.0103157.s001.pdf]

Supporting Results and Discussion

Characterization of the TatA oligomeric states

The oligomeric state of TatA in DPC micelles varies when the DPC concentration changes. For full-length wt-TatA, we observed a single set of cross peaks corresponding to the monomeric form (1st set) at DPR over 200. This result is similar to those reported by Rodriguez *et al* [1], in which they showed that the average oligomeric state is 2.2 at DPR of 250:1 by analytical centrifuge while the NMR spectroscopy display signals of a monomeric form. When the DPR decrease to 20, the full-length wt-TatA displays two clearly distinguishable sets of peaks (1st and 2nd sets). For some residues, a 3rd set of peak was also observable but rather weak (Figure S3). However, the signals of the 3rd set are too scarce and too weak in our case to allow more detailed analysis. When we tried to further decrease the DPR by buffer exchange, we observed that the 3rd set became dominant, while the 2nd set disappeared (Figure S4). However, the NMR sample under this condition was unstable and precipitated after the HSQC experiments, and the exact DPR was difficult to determine.

To overcome the problem of spectral overlap, we repeated the oligomeric state characterizations using a truncated TatA mutant ($TatA_{1-55}$). Judging by the positions of glycine cross-peaks and backbone NH chemical shifts of residues in the hinge region (Figure S5), the 3rd set of signals is most similar to those of the '9-mer' TatA complex observed by Rodriguez *et al* [1]. Moreover, we observed that the truncated variant shows a somewhat different DPR-dependent oligomerization tendency as compared to the full-length protein. Briefly, the $TatA_{1-55}$ sample aggregates more easily compared to full-length TatA. For example, at DPR of about 20, the full-length TatA shows a peak volume distribution for states 1st:2nd:3rd of ~ 3.3:2.4:1, with the monomeric state in the largest population (Figure S3). However, for $TatA_{1-55}$, at even higher DPR (~30), the peak volume distribution of the 1st:2nd:3rd set is 0.75:1:1, with the higher oligomeric states dominant (Figure S5). This result suggests that the unstructured C-terminus (residue 56-89) may have negative effects on TatA oligomerization in DPC micelles. Nevertheless, the patterns

and positions of the three peak sets are essentially similar for $TatA_{1-55}$ and full-length TatA. Therefore, we used the $TatA_{1-55}$ sample for further PRE and dynamics measurements to determine the oligomeric state of the 2nd set of peaks.

Oligomeric state determination of 2nd set of peaks by backbone ^{15}N dynamics

Backbone ^{15}N dynamics data were collected for $TatA_{1-55}$ at DPR of 70. Under this condition, the spectra showed two sets of peaks. The R_2/R_1 ratios were calculated for both the 1st and 2nd sets of peaks, and the results are shown in Figure 1B. The average R_2/R_1 ratio of residues on the TMH region is 26 for the 1st set, and 52 for the 2nd set. By assuming a protein-DPC micelle with a spherical shape, the rotational correlation time could be estimated using the formula $\tau_c \approx \frac{1}{4\pi\nu_N}\sqrt{6\frac{R_2}{R_1}-7}$ [2]. In combination with the Stoke's law $\tau_c = \frac{4\pi\eta r^3}{3kT}$, the diameter of the globular complex can be back calculated using the R_2/R_1 ratio. The average R_2/R_1 ratio of the 1st set suggests a protein-DPC complex with a diameter of 51.4 Å, which fits well with a single transmembrane helix surrounded by ~ 54 DPC molecules [3] (Figure S6). The average R_2/R_1 ratio of the 2nd set suggests a protein-DPC complex with a diameter of 57.4 Å, corresponding to a ~3 Å increase in radius compared to the 1st set. A dimeric model containing two transmembrane helices surrounded by the DPC molecules suggests an average increase of 2.8 Å in radius, whereas a tetrameric model would result in a more significant increase (~6.6 Å) in radius (Figure S6). Therefore, the observed backbone dynamics data of the 2nd set of peaks supports the formation of dimers instead of higher oligomeric states.

Furthermore, we constructed an N-terminal disulfide-linked dimer 'd-MCG-$TatA_{1-55}$'. The manually introduced linkage helps hold two TatA molecules together, enabling the observation of the 2nd peak set at relatively high DPR. We measured the backbone dynamics data of the d-MCG-$TatA_{1-55}$ sample at DPR ~100 (Figure S7). The results showed that the average R_2/R_1 for residues in the TMH (~ 49) is similar to the value observed for the 2nd set of peaks of the $TatA_{1-55}$ sample.

Oligomeric state determination of 2nd set of peaks by PRE

The inter-subunit PRE effects measured using a mixed sample of spin-labeled (TatA$_{1-55}$-I12C-MTSL) and ^{15}N-labeled TatA$_{1-55}$ could be used to obtain information on the oligomerization state of the observed peaks. As illustrated in Figure S8, the sideway-pointing spin label would only affect a fraction of the observable NMR signals and cause a reduction of the peak intensities recorded. Based on a dimeric model, the expected signal reduction is ~ 25%. In contrast, the signal intensities are expected to show 40-50% reduction in the cases of trimeric or tetrameric models. The experimental results showed an average 25% signal decrease, which is in agreement with the dimeric model. A similar analysis of the TatA$_{1-55}$-I11C-MTSL sample arrives at the same conclusion that the PRE effects are better correlated with the dimeric model.

References

1. Rodriguez F, Rouse SL, Tait CE, Harmer J, De Riso A, et al. (2013) Structural model for the protein-translocating element of the twin-arginine transport system. Proc Natl Acad Sci U S A 110: E1092-1101.
2. Q. Teng (2005) Structural Biology: Practical NMR Applications. Springer
3. Tieleman DP, van der Spoel D, Berendsen HJC (2000) Molecular dynamics simulations of dodecylphosphocholine micelles at three different aggregate sizes: Micellar structure and chain relaxation. J Phys Chem B 104: 6380-6388.

Supporting Table

Table S1. Protein samples and conditions used in this study.

Experiments	Sample	TatA conc. /mM	DPC conc. /mM[a]	DPR[b]	Signals[c]
Monomeric structure determination	TatA	1	300	300	1st
Characterization of the oligomeric state	TatA	0.4	9	20	1st, 2nd, 3rd
Dynamics	TatA	0.5	~100	200	1st
Dynamics	$TatA_{1\text{-}55}$	0.9	64	70	1st , 2nd
Dynamics	d-MCG-$TatA_{1\text{-}55}$	1[d]	97	97	
PRE (I12C-MTSL)	$TatA_{1\text{-}55}$	0.5 (0.5)[e]	40	40	1st, 2nd
PRE (I11C-MTSL)	$TatA_{1\text{-}55}$	0.5 (0.5)[e]	45	45	1st, 2nd
Dimeric structure (filter experiments)	d-MCG-TatA	0.5 (1)[f]	130	86	

a. The DPC concentration was determined by the ^{31}P spectra compared with buffer signals.

b. DPR (detergent : protein molar ratio)

c. Dominant set of signals in NMR spectrum.

d. The protein concentration was calculated based on monomeric TatA molecular weight.

e. The concentration noted outside and inside the brackets corresponds to ^{15}N-labeled and MTSL-labeled components, respectively.

f. The concentration noted outside and inside the brackets corresponds to ^{15}N-labeled and unlabeled components, respectively.

Supporting Figures

Figure S1

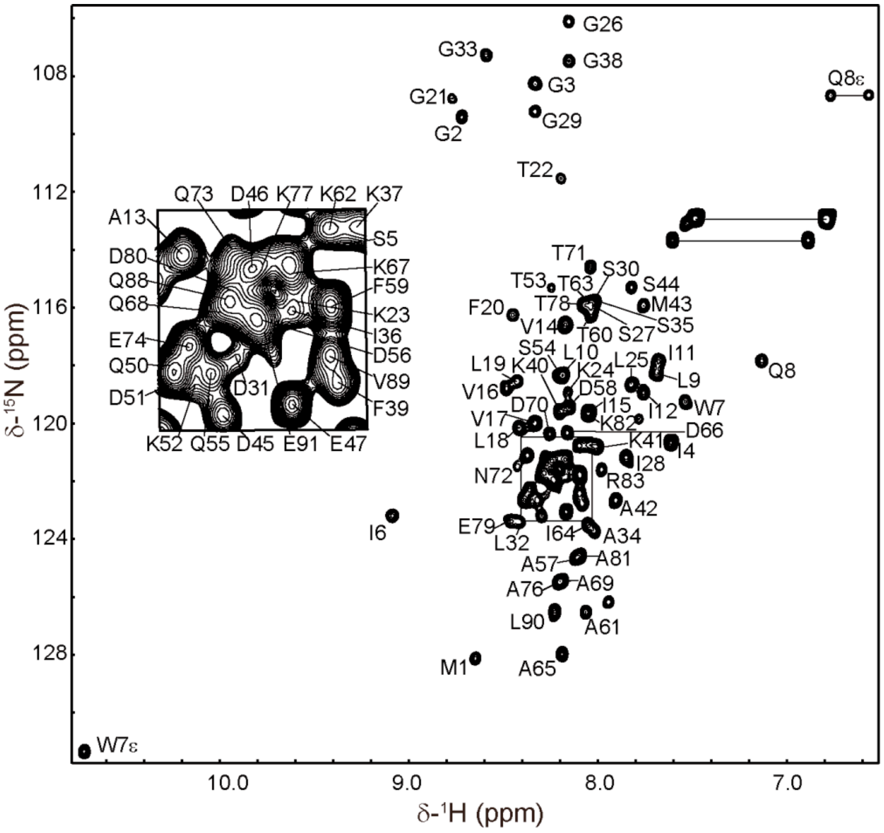

Figure S1. 2D ^{1}H-^{15}N HSQC spectrum of TatA. 2D ^{1}H-^{15}N HSQC spectrum of TatA in DPC micelles annotated with the backbone assignments. The spectrum was collected on a Bruker Avance 800 MHz spectrometer (with a cryo-probe) at 35 °C. The assignments are labeled with the one-letter amino acid code and residue number. The side chain NH_2 peaks of Asn and Gln are connected by horizontal lines. The asterisk indicates residues from the His-tag.

Figure S2

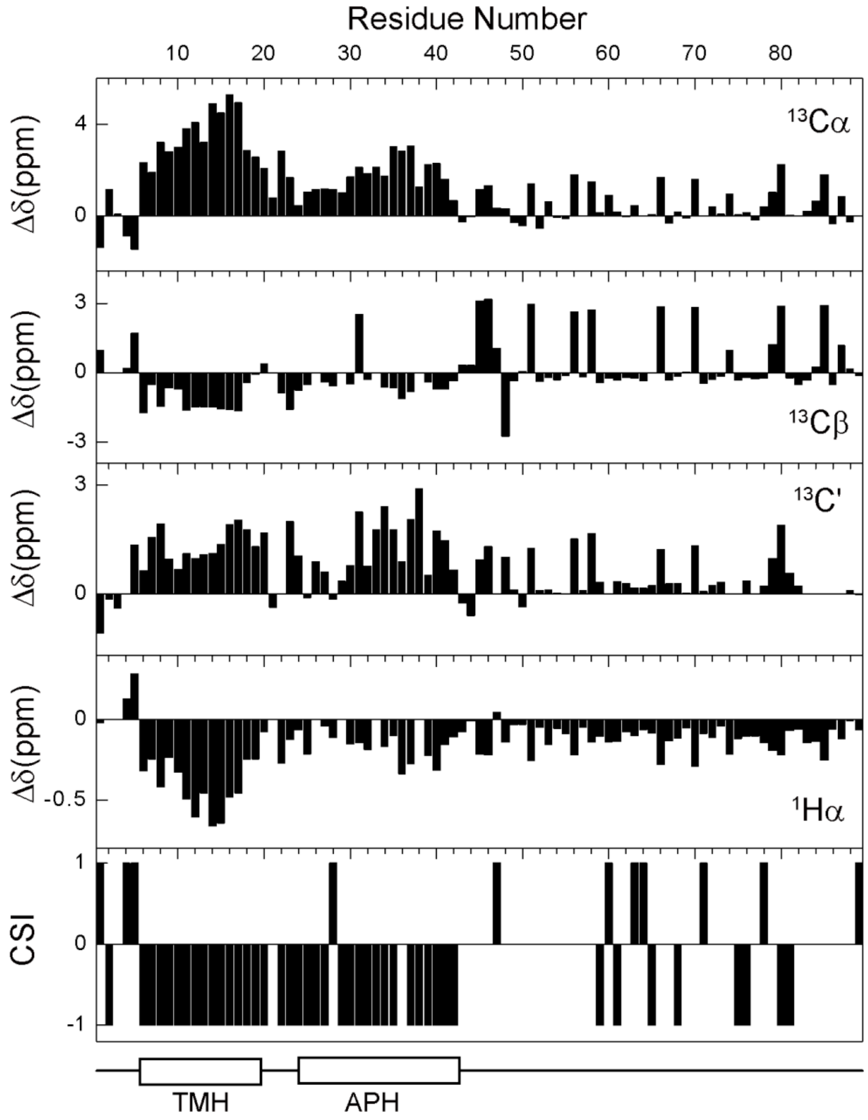

Figure S2. Secondary structures of *E. coli* TatA. Secondary chemical shifts for $^{13}C_{\alpha}$, $^{13}C_{\beta}$, $^{13}C'$ and $^{1}H_{\alpha}$ resonances versus residue numbers and the consensus chemical shift index (CSI) plot of TatA in DPC micelles. The values of CSI for β-strand, α-helix and random coil are 1, -1 and 0, respectively.

Figure S3

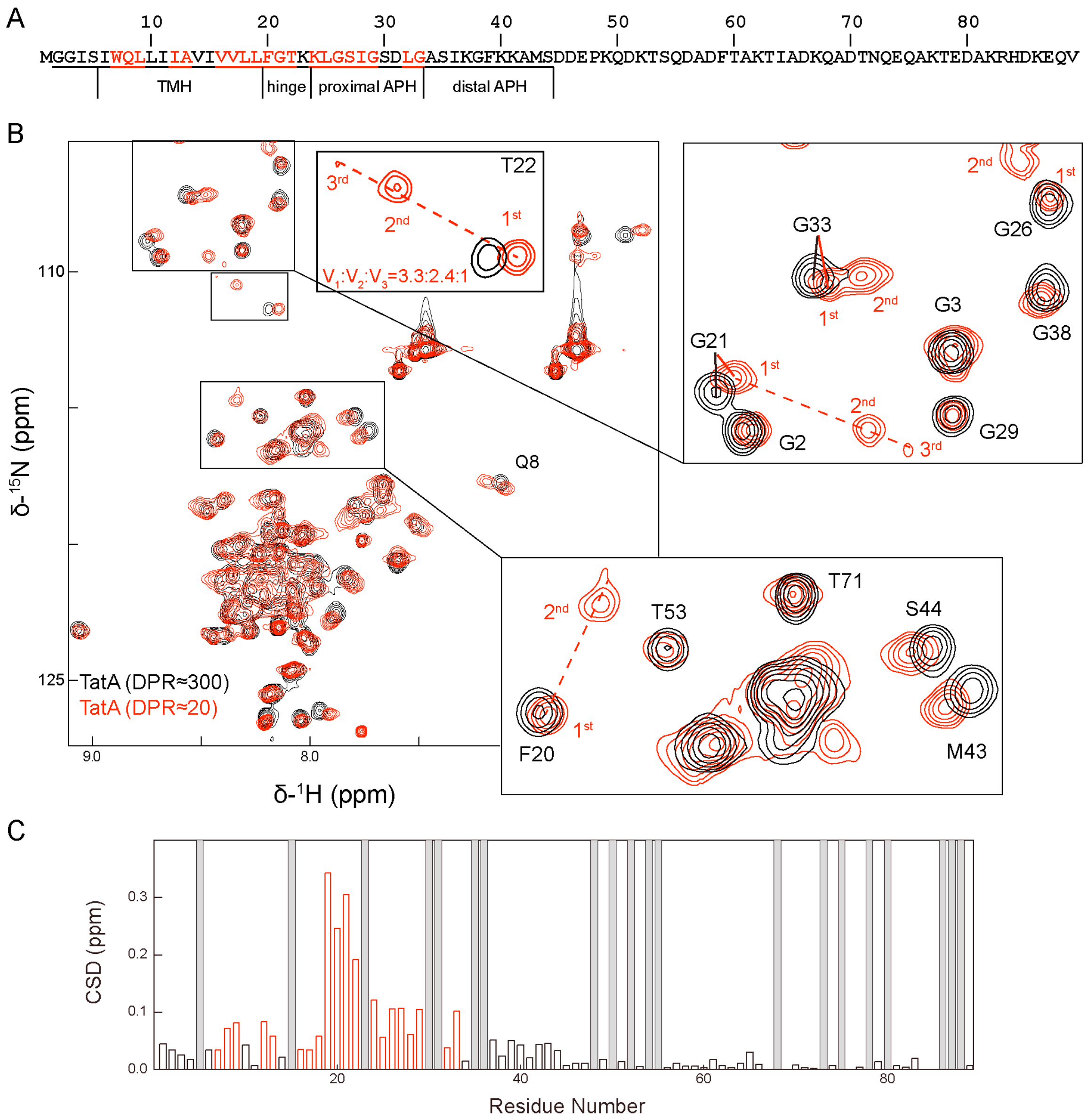

Figure S3. DPR-dependent spectral changes of full-length TatA. (A) The protein sequence of TatA is shown at the top to show the region displaying DPR-dependent chemical shift perturbations (underlined) and residues that have multiple sets of peaks (colored in red). (B) ^{1}H-^{15}N HSQC spectrum of full-length TatA at DPR 300 (black) and 20 (red) with dashed lines indicating the signals from the same residues. The population ratios of different conformers were calculated based on peak volumes (V_1:V_2:V_3). (C) The composite chemical shift differences

between the two peak sets calculated using the formula $CSD = \sqrt{\Delta\delta_H^2 + (\Delta\delta_N / 6.5)^2}$. The red bars represent the residues with multiple sets of signals and the CSD was calculated between the 1st and 2nd peak sets. The black bars represent residues that show only chemical shift perturbations. Grey background represents residues that could not be analyzed.

Figure S4

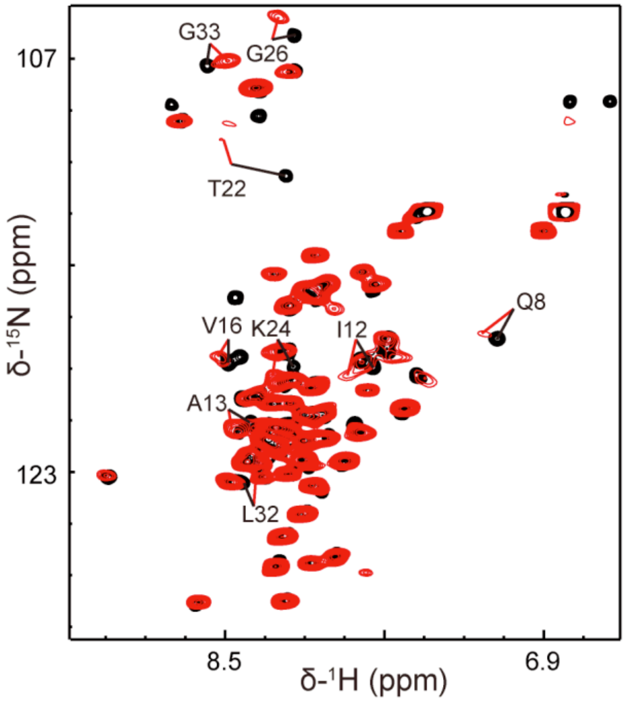

Figure S4. Characterization of TatA at very low DPR. ^{1}H-^{15}N HSQC spectrum of full-length TatA at DPR of 300 (black) and an extremely low DPR (< 20, red). Representative residues are labeled with black and red lines.

Figure S5

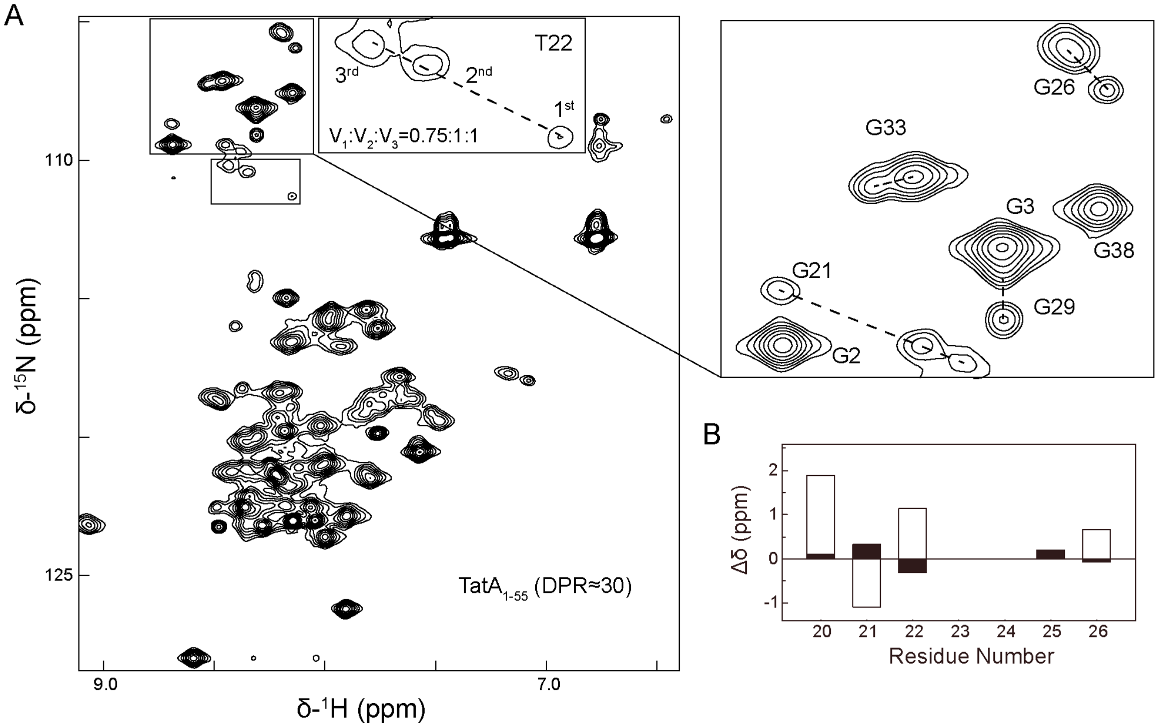

Figure S5. Characterization of $TatA_{1-55}$ at DPR of 30. (A) ^{1}H-^{15}N HSQC spectrum of $TatA_{1-55}$ at DPR of 30 with dashed lines indicating the signals from the same labeled residues. The population ratios of different conformers were calculated based on peak volumes. (B) Chemical shift perturbations between the 1st and 3rd peak sets of residues in the hinge region. Open and filled bars correspond to the chemical shift changes of backbone ^{15}N and ^{1}HN atoms, respectively.

Figure S6

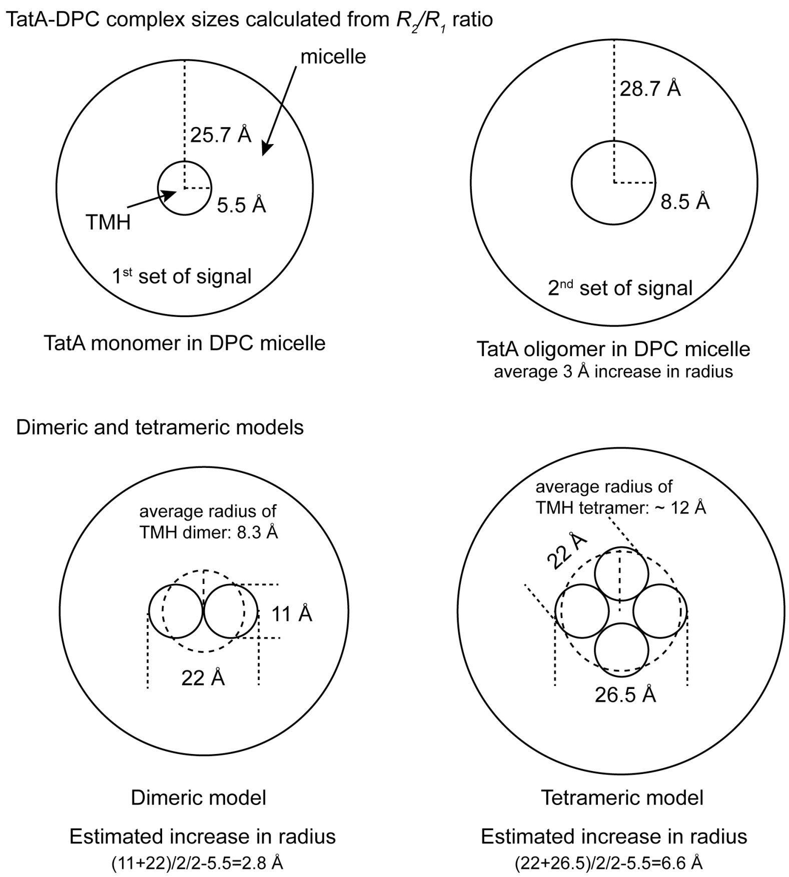

Figure S6. Estimation of the TatA-DPC complexes sizes based on backbone dynamics. The upper panel shows the 2D illustrations of back-calculated sizes of TatA-DPC complexes based on the 1st (upper left) and 2nd sets of peaks (upper right). The data of the 1st set corresponds well to a monomeric model (upper left), whereas the 2nd set exhibits a 3 Å increase in radius compared to the 1st set. The lower panel shows schematic models of a dimeric TatA-DPC complex and a tetrameric TatA-DPC complex. The averaged radius contributed by THM is estimated to be 8.3 Å or 12 Å based on the dimeric or tetrameric models, and would result in an apparent radius increase of 2.8 Å and 6.6 Å respectively. The diameter of a single helix is assumed to be 11 Å in all calculations. The experimental data of the 2nd set fits better to the dimeric model.

Figure S7

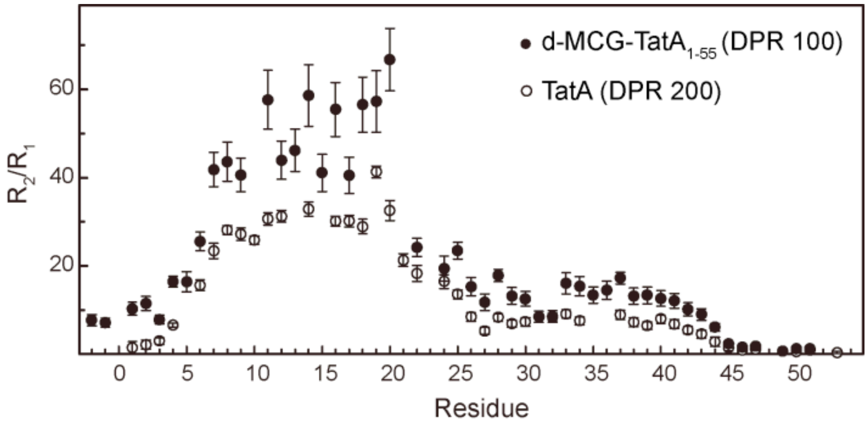

Figure S7. Backbone dynamics of d-MCG-TatA$_{1\text{-}55}$. The backbone ^{15}N relaxation parameter R_2/R_1 values of d-MCG-TatA$_{1\text{-}55}$ at DPR of 100 (filled circle) in comparison with full-length TatA (open circle). The N-terminal MCG-tripeptide extension is numbered as residue -3, -2 and -1 respectively. Residues in the C-terminus of full-length TatA are not shown.

Figure S8

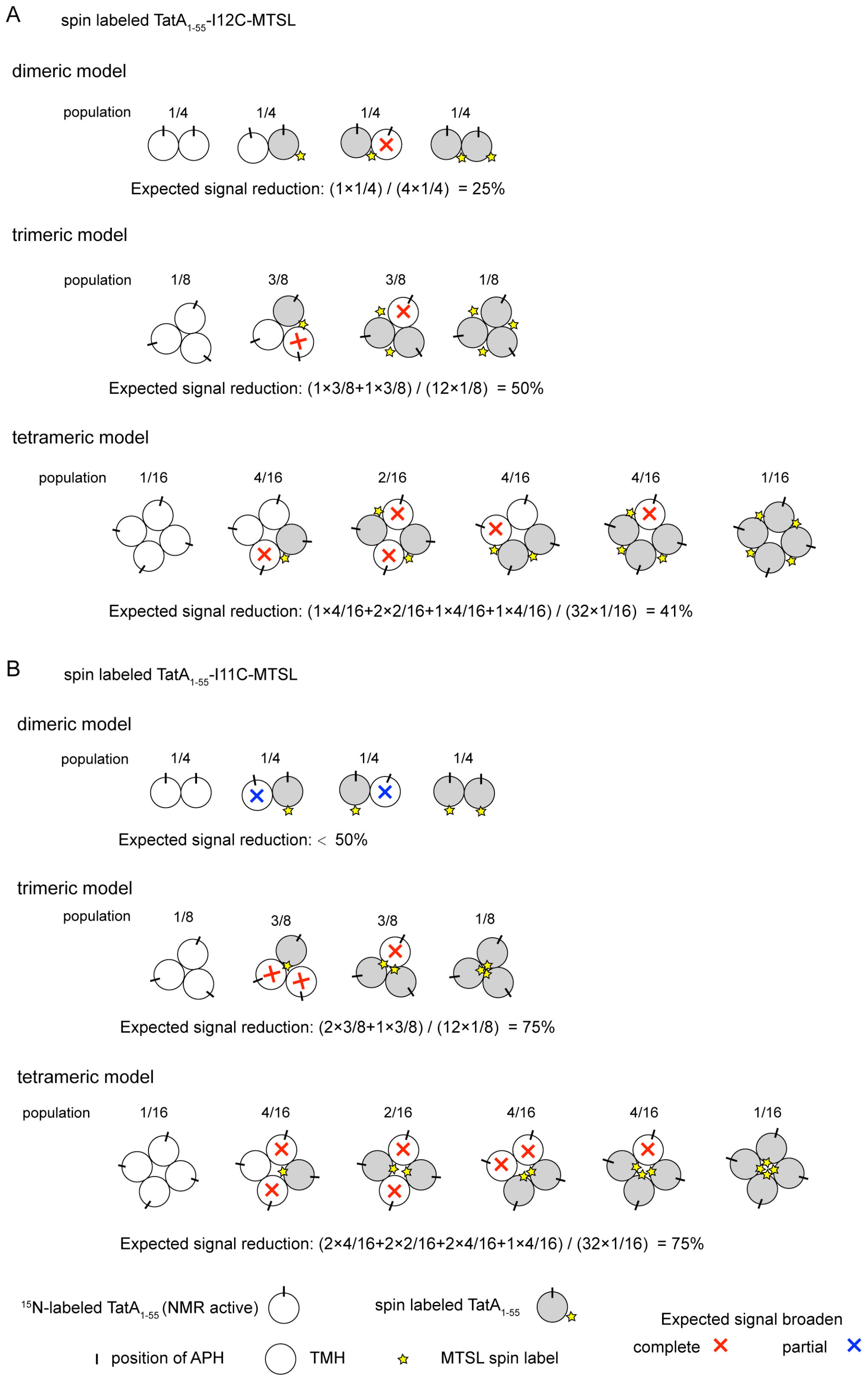
A
spin labeled TatA$_{1-55}$-I12C-MTSL
dimeric model
population 1/4 1/4 1/4 1/4
Expected signal reduction: (1×1/4) / (4×1/4) = 25%
trimeric model
population 1/8 3/8 3/8 1/8
Expected signal reduction: (1×3/8+1×3/8) / (12×1/8) = 50%
tetrameric model
population 1/16 4/16 2/16 4/16 4/16 1/16
Expected signal reduction: (1×4/16+2×2/16+1×4/16+1×4/16) / (32×1/16) = 41%
B
spin labeled TatA$_{1-55}$-I11C-MTSL
dimeric model
population 1/4 1/4 1/4 1/4
Expected signal reduction: < 50%
trimeric model
population 1/8 3/8 3/8 1/8
Expected signal reduction: (2×3/8+1×3/8) / (12×1/8) = 75%
tetrameric model
population 1/16 4/16 2/16 4/16 4/16 1/16
Expected signal reduction: (2×4/16+2×2/16+2×4/16+1×4/16) / (32×1/16) = 75%
^{15}N-labeled TatA$_{1-55}$ (NMR active)
spin labeled TatA$_{1-55}$
Expected signal broaden
complete
partial
position of APH
TMH
MTSL spin label

Figure S8. Illustration of inter-subunit PRE effects of different TatA oligomerization models. Calculation of the population distribution of different oligomeric species and inter-subunit PRE effects using dimeric, trimeric and tetrameric models for paramagnetic labeling on the inter-subunit interface (panel A, $TatA_{1-55}$-I12C-MTSL) or the opposite side of APH (B, $TatA_{1-55}$-I11C-MTSL). The ^{15}N-labeled and spin-labeled samples are mixed at 1:1 molar ratio, and the ^{15}N-labeled sample is the only source generating observable NMR signals. Small yellow star designates the position of the MTSL label, and red and blue crosses indicate expected complete or partial signal broadening by the PRE effect. The expected signal reduction ratio is calculated for each model. The experimental results obtained at DPR ~ 40 for MTSL labeling at either position 12 or 11 best correlate with the dimeric model.

Figure S9

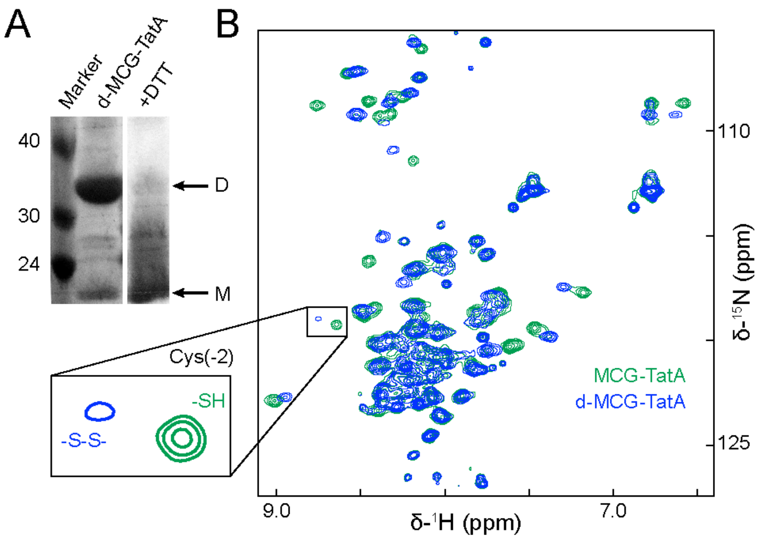

Figure S9. Formation of disulfide-linked d-MCG-TatA dimer. (A) SDS-PAGE spectra of freshly eluted d-MCG-TatA from Ni-NTA and DTT reduced MCG-TatA. Positions of dimer and monomer are labeled. (B) HSQC spectra of d-MCG-TatA and MCG-TatA showing the different positions of the residue cysteine (C-2) in the oxidized and reduced states.

Figure S10

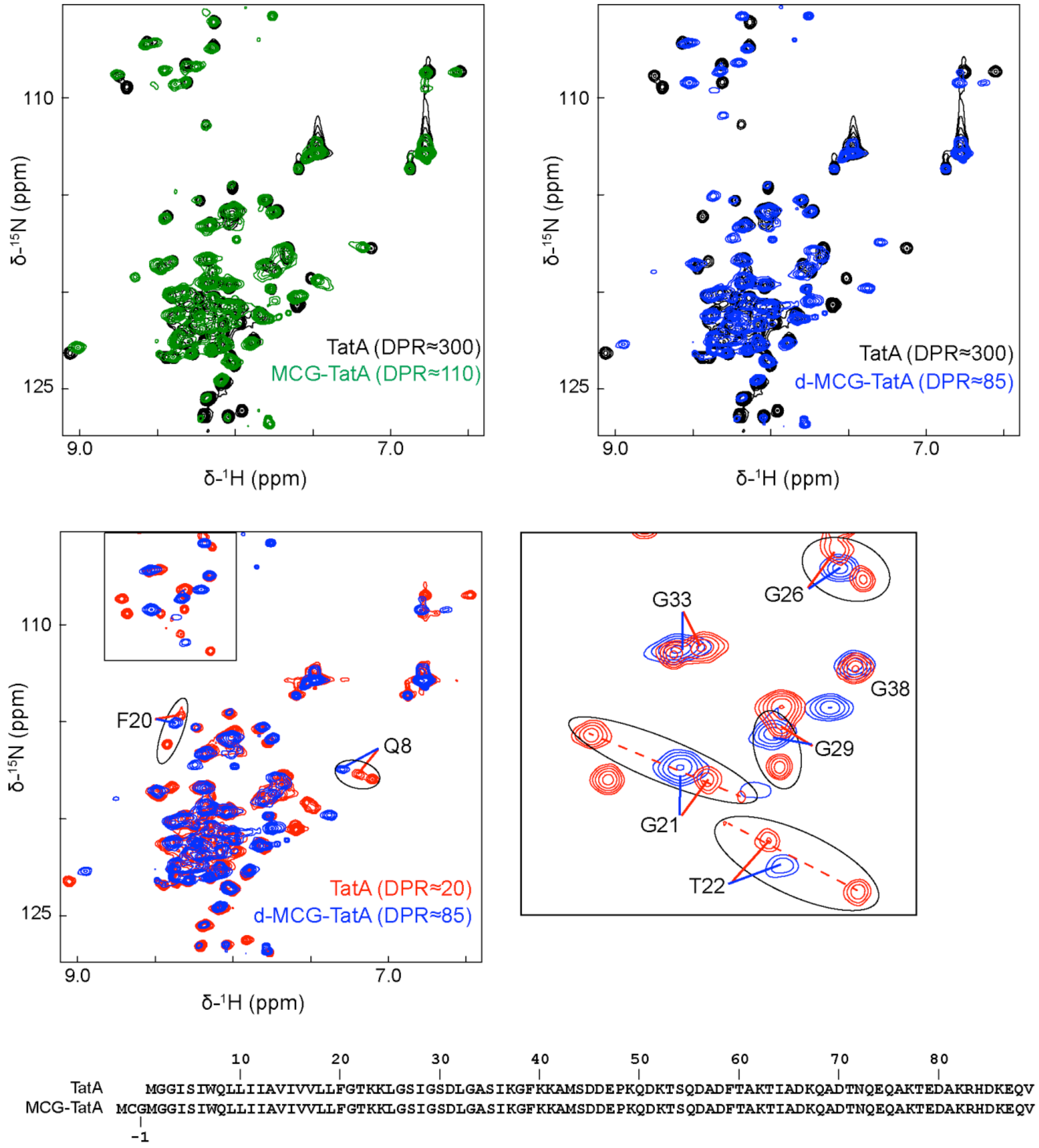

Figure S10. Spectral comparisons of MCG-TatA, d-MCG-TatA and wt-TatA. ^{1}H-^{15}N HSQC spectra of full-length TatA at DPR of 300 (black) and 20 (red) in comparison with MCG-TatA (DPR 110, reduced state) and d-MCG-TatA (DPR 85, oxidized state). The red and blue lines indicate peaks of d-MCG-TatA and the 2nd peak set in TatA (DPR 20), respectively. Representative peaks from the same residue are grouped together and labeled. Apart from

residues 1-8 that are most significantly affected by the N-terminal extension, the other residues show chemical shift differences less than 0.04 ppm for protons and 0.5 ppm for nitrogens between d-MCG-TatA and the 2nd peak set in TatA. The primary sequences of wt-TatA and MCG-TatA are shown at the bottom.

Figure S11

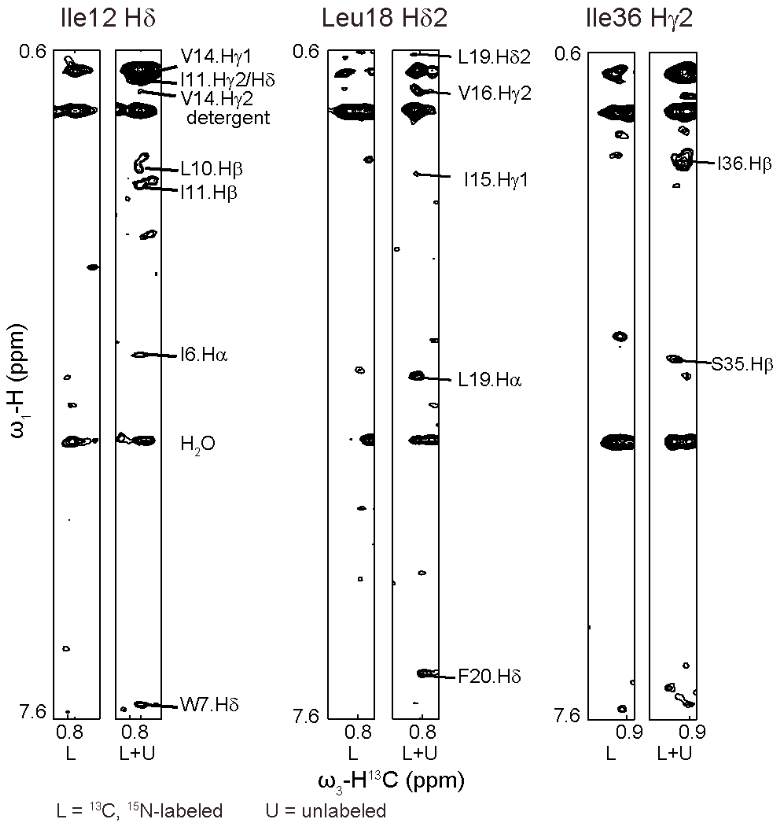

Figure S11. Verification of inter-subunit NOEs. Representative strips from the 3D $^{13}C/^{15}N$-filtered (ω_1), ^{13}C-edited (ω_3) NOESY-HSQC spectra (mixing time 300 ms) of uniformly labeled dimer (left panels) and mixed dimer (right panels). Assignments for inter-subunit NOE cross peaks are labeled in the right panels, whereas no signals are observable in the corresponding positions in the control sample (left panels). The strong peaks present in the control experiment (left panels) originate from detergent and water signals.

Figure S12

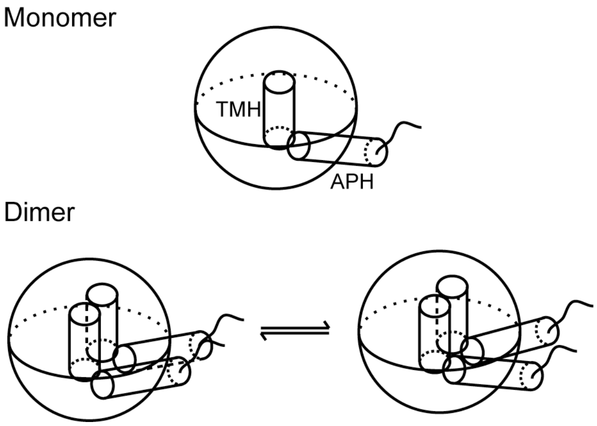

Figure S12. A schematic model of the TatA dimerization.
